# Non-canonical role for the ataxia-telangiectasia-Rad3 pathway in STAT3 activation in human multiple myeloma cells

**DOI:** 10.1007/s13402-023-00817-6

**Published:** 2023-05-01

**Authors:** Lin Li, Xiaoyan Hu, Jewel Nkwocha, Kanika Sharma, Maciej Kmieciak, Hashim Mann, Liang Zhou, Steven Grant

**Affiliations:** 1 Division of Hematology/Oncology, Department of Medicine, Virginia Commonwealth University, P.O. Box 980035, Richmond, VA 23298 USA; 2 Massey Cancer Center, Virginia Commonwealth University, Richmond, VA USA; 3 Department of Translational Medicine, Asklepios BioPharmaceutical, Inc., Durham, NC USA

**Keywords:** ATR, Myeloma, STAT3, Apoptosis

## Abstract

**Purpose:**

The goal of this study was to characterize the relationship between ATR and STAT3 interactions in human multiple myeloma (MM) cells.

**Methods:**

Various MM cell lines, including IL-6-dependent cells were exposed to ATR inhibitors and effects on STAT3 Tyr705 and Ser727 were monitored by WB analysis and ImageStream analysis. Parallel studies examined induction of cell death, STAT3 DNA binding activity, and expression of STAT3 downstream targets (BCL-X_L_, MCL-1, c-MYC). Validation was obtained in ATR shRNA knock-down cells, and in cells ectopically expressing BCL-X_L_, MCL-1, or c-MYC. Analogous studies were performed in primary MM cells and in a MM xenograft model.

**Results:**

Multiple pharmacologic ATR inhibitors inhibited STAT3 Tyr705 (but not Ser727) phosphorylation at low uM concentrations and down-regulated BCL-X_L_, MCL-1, c-MYC in association with cell death induction. Compatible results were observed in ATR shRNA knock-down cells. Cell death induced by ATR inhibitors was significantly attenuated in cells ectopically expressing constitutively active STAT3, BCL-X_L_, MCL-1, or c-MYC. Concordant results were observed in primary human MM cells and in an in vivo MM xenograft model.

**Conclusions:**

Collectively, these findings argue for a non-canonical role for the ATR kinase in STAT3 activation in MM cells, and suggest that STAT3 inactivation contributes to the lethal actions of ATR inhibitors in MM.

**Supplementary Information:**

The online version contains supplementary material available at 10.1007/s13402-023-00817-6.

## Introduction

The ATR (ataxia telangiectasia and Rad3-related) kinase is a serine-threonine kinase that plays a critical role in the DNA damage response (DDR) to single-strand DNA breaks and replication stress [1]. Activation of ATR by such stimuli activates the Chk1 kinase at Ser_327_ and Ser_345_ [2], leading to phosphorylation and subsequent degradation of the CDC25A-C phosphatases [3]. These events culminate in phosphorylation and inactivation of CDK2 and CDK1 (cdc2), thereby triggering the intra-S-phase and G_2_M checkpoints, thereby permitting cells to repair sub-lethal DNA damage [4]. Conversely, disruption of ATR e.g., by pharmacologic ATR inhibitors in cells that have sustained single-strand breaks or which experience stalled replication forks results in inappropriate or premature mitotic entry culminating in mitotic catastrophe and cell death [5]. These considerations have prompted the development of multiple ATR inhibitors which have been shown to potentiate the anti-tumor activity of diverse genotoxic agents including ionizing radiation and cytotoxic drugs [6]. Notably, multiple myeloma (MM), a malignant and generally incurable disorder of mature plasma cells, has been shown to be particularly susceptible to replication stress and ATR inhibition [7].

Deregulation of the STAT3 transcription factor has been linked to the genesis of diverse tumor types, and has also been implicated in tumor migration, metastasis, and refractoriness to both chemotherapeutic agents as well as microenvironmental/stromal cell forms of resistance [8, 9]. STAT3 activation plays a particularly important role in the development of MM and protecting MM cells from various therapeutic strategies, including those targeting the IL-6 pathway [10]. However, like most transcription factors, STAT3 has proven very difficult to target directly with small molecules, prompting the search for alternative approaches such as decoy oligonucleotides or PROTACs [11]. In this context, STAT3 is subject to diverse post-translational modifications, including acetylation, methylation, and phosphorylation [12]. The latter include phosphorylation of the transactivation domain (TAD) at Tyr_705_, involved in STAT3 dimerization and nuclear transport, and at Ser_727_, implicated in DNA binding and transactivation [8]. Indeed, the STAT3 inhibitory activity of several small molecule inhibitors has been related to disruption of STAT3 Tyr_705_ phosphorylation [13].

In addition to its effects on Chk1 activation, ATR modulates other components of the DDR, including stabilization of DNA replication forks and promotion of DNA repair [14]. Currently, however, little information exists linking ATR activation, directly or indirectly, to the STAT3 pathway. Very recently, we reported that the Chk1 kinase was directly involved in STAT3 Tyrosine_705_ phosphorylation and resulting activation, and that pharmacologic Chk1 inhibitors potently disrupted this process while interrupting STAT3 signaling [15]. However, because Chk1 inhibitor development is currently largely inactive, it would be important to determine if ATR inhibitors might act similarly. Here we describe a novel non-canonical function of ATR inhibition in MM cells e.g., disruption of STAT3 signaling, and provide support for a functional role for STAT3 inactivation in the lethal effects of ATR inhibitors in this setting.

## Materials and methods

### Cell lines and reagents

Human MM cell lines U266, OPM2, RPMI8226, KAS-6/1 (1 ng/ml IL-6) cells were maintained in RPMI-1640 supplemented with 10% fetal bovine serum and penicillin–streptomycin. Bortezomib-resistant cells, U266/PS-R and RPMI8226/V10R were established and maintained as described previously [16]. For information regarding the ATR inhibitors Bay1895344 (BAY), AZD6738 (AZD), VE-822 (VE), the potent ATR inhibitor M1774 (EMD Serono, Inc.), Atovaquone (AQ), other reagents, and kits and plasmids, see Supplemental Table 1.

### Analysis of cell death

Loss of mitochondrial membrane potential and cell death were assessed by double staining with 3,3-dihexyloxacarbocyanine (DiOC6) and 7-AAD as before [17].

### Cell viability assay

Cell proliferation was determined by CellTiter ‐Glo luminescence cell viability assay (G7570; Promega, Madison, WI, US) in accordance to the manufacturer’s instructions.

### Transfection and Virus infection

Lentiviruses were generated in Phoenix cells by transfecting cells with packaging DNA plus TRIPZ Inducible Lentiviral shRNA or lenti-CRISPR vectors. Typically, 2 μg vector DNA, 1.5 μg psPAX2, and 1 μg pMD2.G, 10 μl FuGENE® 6 Transfection Reagent (Promega) were used. FuGENE® 6 Transfection Reagent was first added to serum-free medium (Opti-MEM® I Reduced-serum medium). The solution was mixed and incubated for 5 min, after which DNA was added to the FuGENE® 6 Transfection Reagent/medium, which was then mixed and incubated for an additional 15 min. Mixtures were added to Phoenix cells e.g., 5 × 10^6^ cells seeded in one 10 cm dish one day earlier. Viral supernatant was collected two or three days after transfection, filtered through 0.45 μm membranes, and added to target cells in the presence of polybrene (8 μg/ml, Sigma-Aldrich).

Other transfections were performed followed by FuGENE® 6 instructions as above.

### Quantitative RT-PCR

Total RNA was extracted with the RNeasy Plus Mini Kit (Qiagen 74,134) from MM cell lines. One-microgram of RNA was reverse-transcribed by SuperScript one-step RT-PCR Kit (ThermoFisher 10,928–034) according to the manufacturer’s instructions. Using Taqman Gene Expression Assay probe/primer [Hs00153408 for Myc], cDNAs were amplified in a fluorescence thermocycler (ABI StepOnePlus Real-time PCR System, Applied Biosystems, CA, USA) and were analyzed based on the expression level of GADPH with SDS2.2 software (Applied Biosystems).

### Immunoblotting

Samples were prepared from whole-cell pellets followed by lysis with M-PER™ Mammalian Protein Extraction Reagent (Thermo Scientific, Rockford, IL). Total protein or nuclear protein were quantified using Coomassie Protein Assay Reagent (Pierce ThermoFisher Scientific, Rockford, IL). Equal amounts of protein (20 µg) were separated by SDS-PAGE and electro-transferred onto nitrocellulose membrane. Primary antibodies used were: rabbit anti- cleaved PARP, rabbit anti-STAT3, rabbit anti-phospho-STAT3 (Tyr705), rabbit anti-phospho-STAT3 (Ser727), rabbit anti-γH2A.X, rabbit anti-BCL-X_L_, mouse anti-Chk1, rabbit anti-phospho-Chk1 (Ser296), rabbit anti-cleaved Caspase-3, rabbit anti-c-Myc, rabbit anti-MCL-1, mouse anti-FLAG, mouse anti-GAPDH, rabbit anti-β-actin, and mouse anti-α-tubulin. The secondary antibodies used were goat anti-mouse and anti-rabbit IgG-peroxidase labeled. Primary antibodies were used at 1:1000 dilutions. Second antibodies were used at 1:5000 dilutions. Images were captured with the Odyssey® Fc Imaging Syetem (LI-COR). Images were quantified and analyzed by using ImageJ software.

### STAT3 DNA-binding activity and STAT3 reporter

The DNA binding capacity of STAT3 was determined in MM cell nuclear extracts using the TransAM® STAT3 DNA-binding kit (Active Motif 45,196 and 40,010) according to the suppliers’ instructions. Resultant absorbance at 450 nm that correlates with STAT binding to a consensus DNA sequence was read using the Promega Glomax Multi Detection Plate Reader (Promega). STAT3-luciferase reporters based on IL-6 sis-Inducible Element (SIE). Briefly, 293 T cells were transfected in six-well plates using PEI Max polyethylenimine (Polysiciences 75,800–188). The plasmids co-transfected for STAT3 reporter activity and for transfection normalization were pGL4.47[luc2P/SIE/Hygro] and pGL3-Ren-Luc (Promega), respectively, at 5:1 ratio. After 16 h, cells were replated in 96-well plates for 24 h. STAT3 reporter cells were pretreated with compounds for 1 h including vehicle, BAY, AZD, AQ, then stimulated with IL-6 (10 ng/mL) for 5 h. Firefly and renilla luciferase activities were measured following manufacturer's instructions (Dual-Luciferase Reporter Assay System, Promega PAE1910) using the Promega Glomax Multi Detection Plate Reader (Promega).

### Flow Cytometry using ImageStream

Cells were fixed in 4% paraformaldehyde for 15 min at room temperature, and permeabilized by incubation in 90% methanol on ice for 30 min. For primary samples, cells were incubated in 1:10 CD-138-PE (Miltenyi Biotec Cat# 130–117-395) for ten minutes at 4 °C before fixation. Then cells were incubated with primary antibodies for 1 h at room temperature: 1:200 anti-phospho-STAT3(Tyr705) (Cell Signaling Technology Cat# 9145, RRID: AB_2491009), and secondary antibody 1: 500 APC (Invitrogen Cat# 31,984) at room temperature, in the dark for 30 min. Cells were also stained with DAPI 1:100 for 5 min before imaging. The cells were washed in 1000 µl of PBS-2%FBS and recovered by centrifugation at 300 g for 5 min; and the incubation buffer for the antibodies was made from 5% BSA and 0.3% TritonX dissolved in PBS (phosphate‐buffered saline). Cells were resuspended in 60 µl of PBS-2%FBS and analyzed with an ImageStream^X^ (Amnis) image flow cytometer.

### Isolation of primary MM cells

Bone marrow samples from MM patients undergoing diagnostic aspirations at the VCU Health Massey Cancer Center were obtained with informed consent and approval of the VCU IRB (IRB #MCC-8712-3A; MCC-02447; MCC-03340). These studies were conducted in accordance with recognized ethical guidelines (e.g., the Declaration of Helsinki).

For MM primary cell analysis, mononuclear cells were isolated from a total of 5 MM patient bone marrows using the Ficoll Histopaque method (#10,771, Sigma-Aldrich, USA). Isolated cells were cultured in flasks containing RPMI1640 medium and 10% FBS at a concentration of 10^6^ cells/ml. They were then exposed (20–24 h) to ATR inhibitors, after which the sub-population of CD138 + cells were analyzed by flow cytometry to monitor expression of p-STAT3 Y705 and DAPI as we have previously described [15].

### Patient-derived conditioned medium (PDCM)

Patient-derived conditioned medium (PDCM) was obtained from a patient-derived stomal cells, as previously described [15].

### Animal studies

All animal studies were performed under protocol AD10000035, approved by our local IACUC, and regulated by VCU’s Animal Care and Use Program, in accordance with AAALAC, USDA, and PHS guidelines. NOD-SCID IL2Rgamma^null^ mice (Jackson Laboratories, Bar Harbor, ME) were subcutaneously injected with 5 × 10^6^ U266 cells into the flank. The ATR inhibitor BAY was prepared in a 6:1:3 (v/v/v) mixture of PEG400, Ethanol and ultrapure water at a concentration of 3.75 mg/ml. When tumors grew to 300 mm^3^, BAY (30 mg/kg) was administrated (p.o.) for 3 days. Control animals received equal volumes of vehicle. Tumors were collected to perform immunoblotting.

### Statistical analysis

Values represent the means ± SD for three separate experiments. The significance of differences between experimental variables was determined using the Student’s t-test. Values were considered statistically significant at *, P < 0.05; **, P < 0.01; ***, P < 0.001.

For additional information and methods, see Supplementary Materials and Methods, and Supplemental Table 1.

## Results

### ATR inhibitors induce apoptosis in parental and bortezomib-resistant myeloma cells

**Fig. 1:**
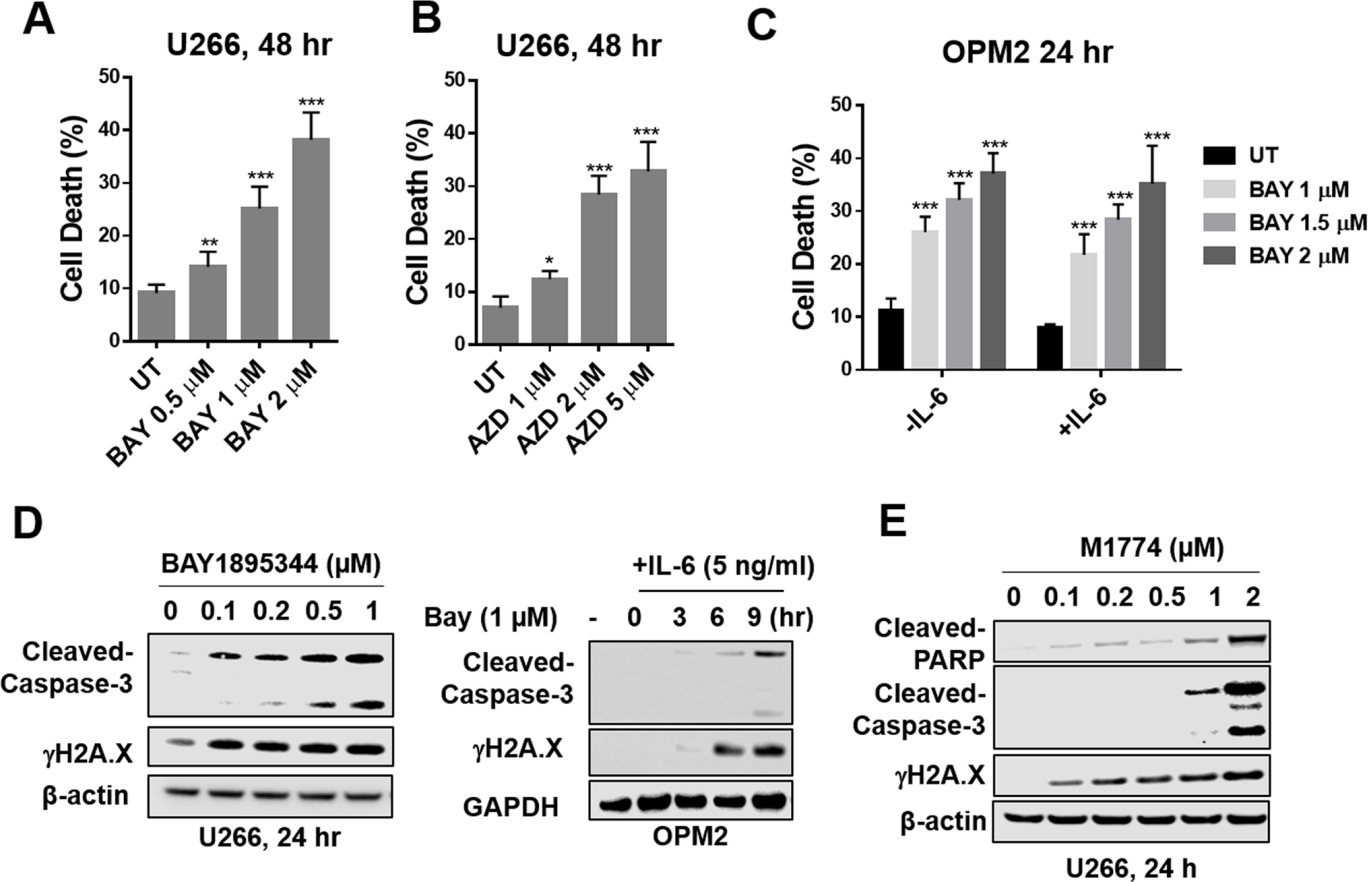
ATR inhibitors induce apoptosis in multiple myeloma cells. (**A**-**C**) U266 and OPM2 cells (± 5 ng/m IL-6) were exposed to the indicated concentrations of Bay1895344 or AZD6738 for 24 or 48 h, followed by flow cytometric analysis of cell death after staining with 7-AAD. Values represent the means ± S.D. for three experiments performed in triplicate. * = *P* < 0.05; ** = *P* < 0.01; *** = *P* < 0.001 significantly greater than values for untreated controls. (**D**-**E**) U266 and OPM2 cells (± 5 ng/m IL-6) were incubated with Bay1895344 or M1774 for the indicated interval, after which γH2A.X and cleavage of caspase-3, or cleavage of PARP were monitored by immunoblotting analysis. β-actin or GAPDH was assayed to ensure equivalent loading and transfer. Results are representative of 3 separate experiments

Exposure of IL-6-independent (U266) or -stimulated (OPM2) cells, the latter cultured in the presence of 5 ng/ml IL-6, to 0.5 to 2 μM concentrations of the ATR inhibitors BAY1895344 or AZD6738 for 24 to 48 h significantly induced apoptosis as determined by 7-AAD uptake by flow cytometry (Fig. 1A-C). Parallel studies demonstrated a time- and concentration-dependent increase in caspase-3 cleavage and γH2A.X formation (Fig. 1D-E), including in U266 cells exposed to the novel ATR inhibitor M1774 (Fig. 1E). Highly bortezomib-resistant PS-R cells (Supplemental Fig. S1A) [18] exhibited modest but statistically significant increases in cell death following exposure to ATR inhibitors (Supplemental Fig. S1B-E). ATR inhibitors (BAY and M1774) significantly induced cell death in multiple other MM cell lines, including H929, KMS11, RPMI8226, Velcade-resistant RPMI8226 (RPMI8226/V10R), and IL-6-dependent KAS/6-1cells (Supplemental Fig. S2). Moreover, western blot analysis revealed that in these lines, ATR inhibitors induced caspase and PARP cleavage as well as down-regulation of STAT3-Y705 phosphorylation, but minimal effects on p-STAT3-S727 (Supplemental Fig. S3). These findings indicate that clinically relevant ATR inhibitors induce cell death in multiple MM cell lines at low and sub-μM concentrations, including in IL-6-dependent and bortezomib-resistant cells.

### ATR inhibitors block tyrosine phosphorylation of STAT3 (p-Y705) and inhibit STAT3 signaling pathways in MM cells

**Fig. 2:**
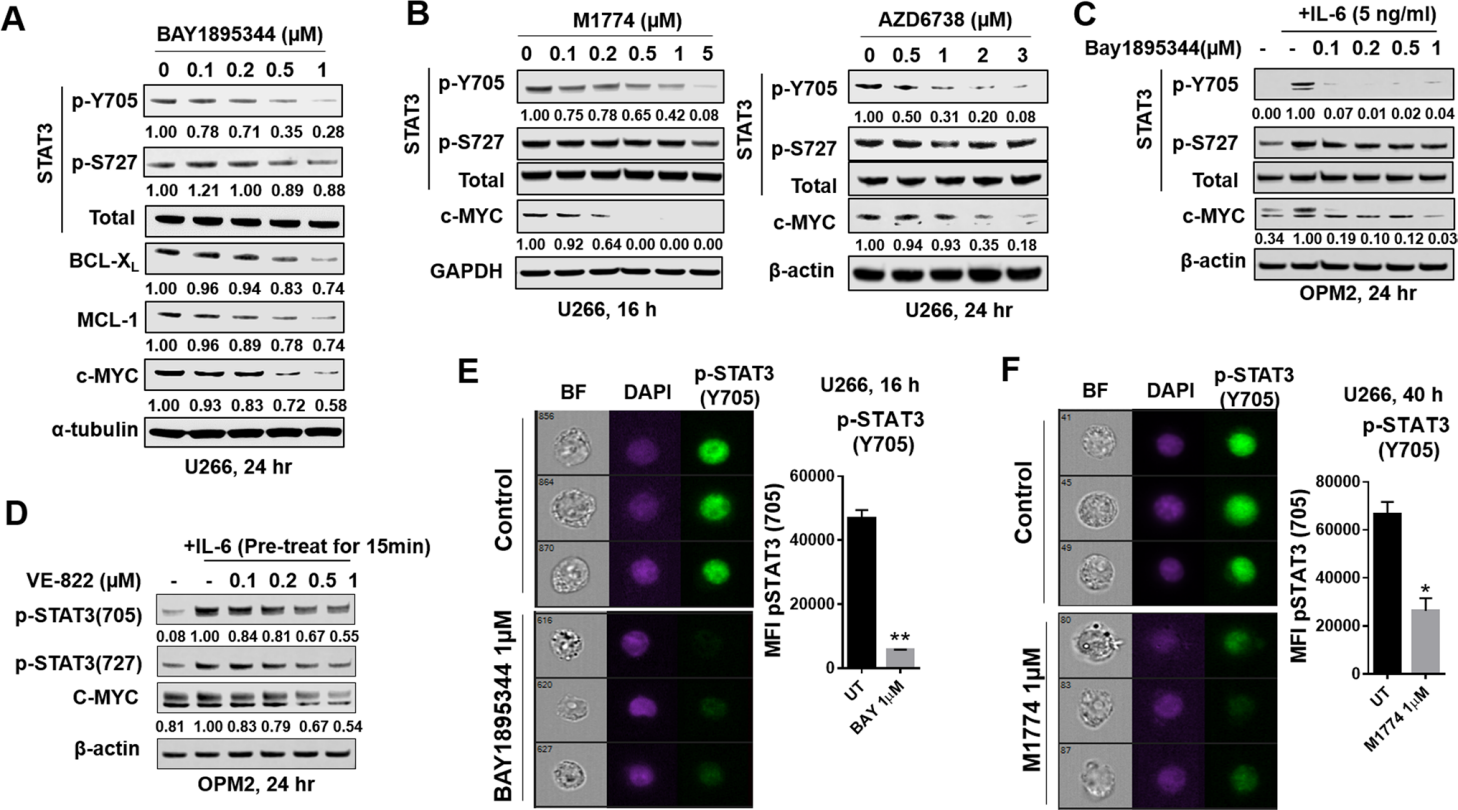
ATR inhibitors block tyrosine phosphorylation of STAT3 (p-Y705) and inhibit STAT3 signaling pathways in MM cells. (**A**-**B**) Western blot analysis of p-Y705 STAT3, p-S727 STAT3, total STAT3, and the STAT3 downstream targets MCL-1, BCL-X_L_, and c-Myc, in U266 cells treated with indicated concentrations of Bay1895344, AZD6738, or M1774 for 16 or 24 h. α-tubulin, β-actin, or GAPDH controls were assayed to ensure equivalent loading and transfer. (**C**-**D**). WB analysis of p- or total-STAT3, and c-MYC in OPM2 cells pre-treated for 15 min with 15 ng/ml IL-6 and exposed to Bay1895344 or VE-822 for 24 h. Images were quantified by densitometry and analyzed using Imagel software. (**E**–**F**) Untreated and treated (Bay1895344 1 µM, 16 h or M1774 1 µM, 40 h) cells were stained with p-Y705 STAT3 and DAPI, and then visualized by ImageStream imaging. Representative cells are shown (BF = brightfield). Histogram of p-Y705 STAT3 intensity and fold-change are shown. Representative data for at least three replicates. *, *P* < 0.05; **, *P* < 0.01 versus untreated controls

Exposure of U266 cells (24 h) to 0.5–1.0 μM BAY markedly diminished expression of STAT3 p-Y705, but had little effect on p-S727 (Fig. 2A). This was accompanied by down-regulation of the STAT3 downstream targets BCL-X_L_, MCL-1, and c-MYC (Fig. 2A). Parallel studies with the ATR inhibitors M1774 and AZD6738 yielded similar results e.g., down-regulation of p-Y705 but not p-S727, and diminished expression of c-MYC (Fig. 2B). BAY and the ATR inhibitor VE-822 [19] exerted comparable effects in IL-6-treated OPM2 cells (Fig. 2C-D). Both BAY and M1774 acted similarly e.g., down-regulated c-MYC, BCL-X_L_, and MCL-1 in highly bortezomib-resistant PS-R cells (Supplemental Fig. S4A-B) as well as in OPM2 cells cultured in the presence of patient-derived stromal cell-conditioned medium (Supplemental Fig. S4C-D). In contrast, ATR inhibitors had little effect on the expression of SOCS1/3, regulators of STAT3 signaling (Supplemental Fig. S5). Finally, significant reductions in the expression of p-STAT3 (Y705) in U266 cells monitored by ImageStream analysis were observed following treatment of cells with BAY or M1774 (Fig. 2E-F). Together, these findings argue that ATR inhibitors block STAT3 p-Y705 phosphorylation and down-regulate STAT3 downstream targets.

### ATR inhibitors block STAT3 activation

**Fig. 3:**
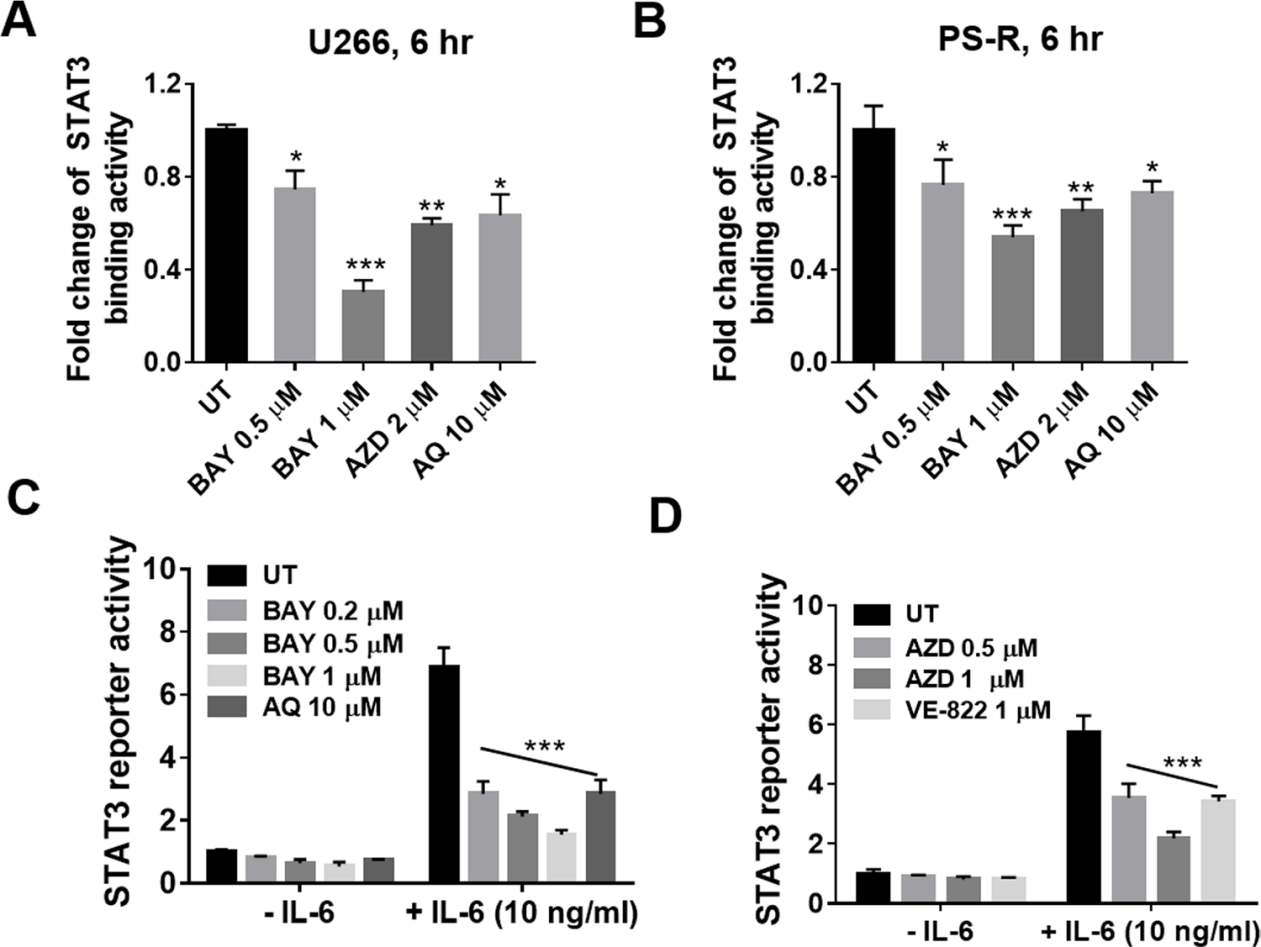
ATR inhibitors block STAT3 activation in multiple myeloma cells. (**A**-**B**), U266 and PS-R cells were treated with the indicated concentrations of Bay1895344, AZD6738, or AQ (Atovaquone) for 6 h. A STAT3 DNA-binding ELISA assay was used to evaluate the DNA binding of STAT3 in cellular nuclear extracts. The relative fold-change (vs untreated controls; UT) is presented. (**C**-**D**), STAT3 reporter (STAT3-Luc) transfected 293 T cells were pre-treated with the indicated concentrations of Bay1895344, AZD6738 or AQ for 1 h, after which they were stimulated with IL-6 (10 ng/mL) for 5 h. Data are presented as median values with standard deviations for triplicates. *, *P* < 0.05; ** = *P* < 0.01; *** = *P* < 0.001, when compared to the UT group cultured in the presence of IL-6

The effects of ATR on STAT3 activation was examined in U266 and bortezomib-resistant cells using a STAT3 DNA-binding ELISA assay. Exposure (6 h) to BAY (1 μM) or AZD (2 μM) in both parental U266 and bortezomib-resistant PS-R cells significantly reduced STAT3-DNA binding activity (Fig. 3A-B). These agents were equally effective in inhibiting STAT3 activity as 5- to tenfold higher concentrations of atovaquone, an anti-parasitic agent shown to induce STAT3 down-regulation in MM and AML cells [20]. In accord with these findings, a Luc-transfected 293 T cell reporter assay demonstrated a marked increase in STAT3 reporter activity with addition of IL-6, and significant reductions by the ATR inhibitors BAY, AZD, and VE-822, as well as a high concentration of atovaquone [20] (Fig. 3C-D). These findings indicate that ATR inhibitors significantly diminish STAT3 activity in MM and other cell types.

### ATR knock-down disrupts STAT3 signaling

**Fig. 4:**
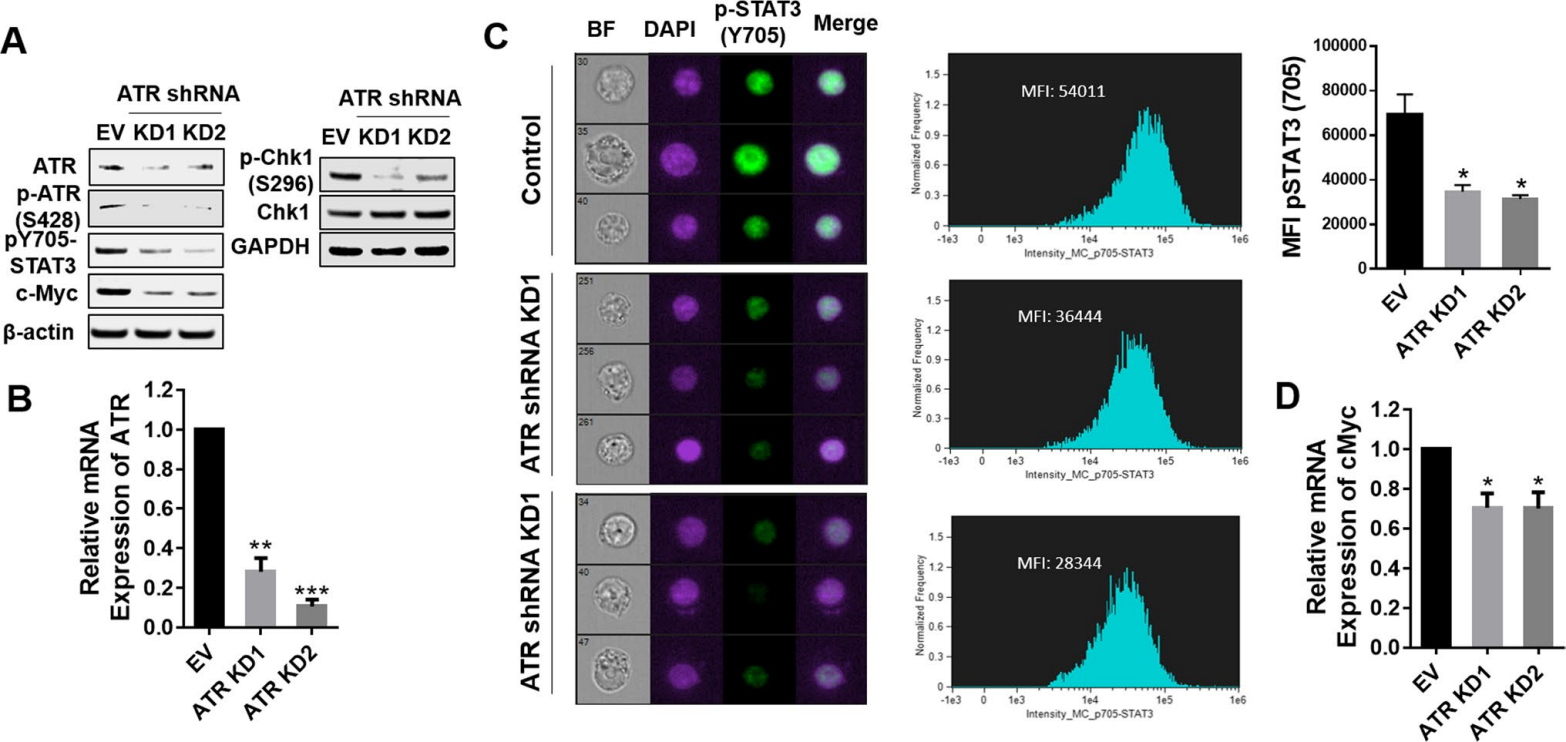
ATR knockdown disrupts STAT3 signaling in multiple myeloma cells. (**A**) U266 cells were transfected with a lentivirus harboring ATR shRNA. Western blot analysis of ATR, p-ATR(S428), p-STAT3 (Y705), c-MYC, p-Chk1 (S296), and Chk1 in U266-EV or ATR shRNA cells is shown. (**B**) Relative mRNA expression of ATR was determined by real-time reverse transcription-PCR analysis. GAPDH served as an internal control. The relative fold change (vs EV) is presented. (**C**) U266-EV or ATR shRNA cells were stained with p-Y705 STAT3 and DAPI, and then visualized by ImageStream, after which representative cells are shown (BF = brightfield). Histogram of p-Y705 STAT3 intensity and-fold change are shown. Representative data for at least three replicates were obtained. (**D**) Relative mRNA expression of c-Myc was determined by real-time reverse transcription-PCR analysis. GAPDH served as an internal control. The relative fold change (vs EV) is presented. *, *P* < 0.05; ** = *P* < 0.01; *** = *P* < 0.001

To validate the above findings through genetic means, U266 ATR knock-down cells were generated by transfecting cells with ATR shRNA. Two knock-down single cell clones were generated (KD1 and KD2) with diminished expression of ATR as well as p-ATR(S428) (Fig. 4A, left panel). KD1/2 cells also displayed diminished expression of STAT3 p-Y705 as well as c-MYC compared to empty-vector controls. In separate studies, KD1 cells exhibited no change in total Chk1 expression, but diminishded expression of p-Chk1 (S296) (Fig. 4A, right panel). RT-PCR analysis of ATR expression demonstrated significant reductions in ATR mRNA levels in ATR knock-down cells compared to empty-vector controls (P < 0.01; Fig. 4B). Furthermore, KD1/2 cells exhibited a sharp decrease in cellular expression of p-STAT3 (Y705) compared to parental cells as reflected by ImageStream analysis (Fig. 4C). Quantitation of p-STAT3 (Y705) expression indicated that these reductions were statistically significant (Fig. 4C, upper right panel; P < 0.05). Finally, RT-PCR analysis of c-Myc expression documented significant reductions in c-Myc mRNA levels in ATR knock-down cells compared to empty-vector controls (P < 0.05; Fig. 4D). Together, these findings indicate that ATR genetic knock-down phenotypically recapitulates the inhibitory effects of pharmacologic ATR inhibitors on STAT3 pY705 inactivation.

### Constitutive STAT3 activation and enforced expression of its downstream targets c-MYC, BCL-XL, MCL-1 attenuate ATR inhibitor lethality in MM cells

**Fig. 5:**
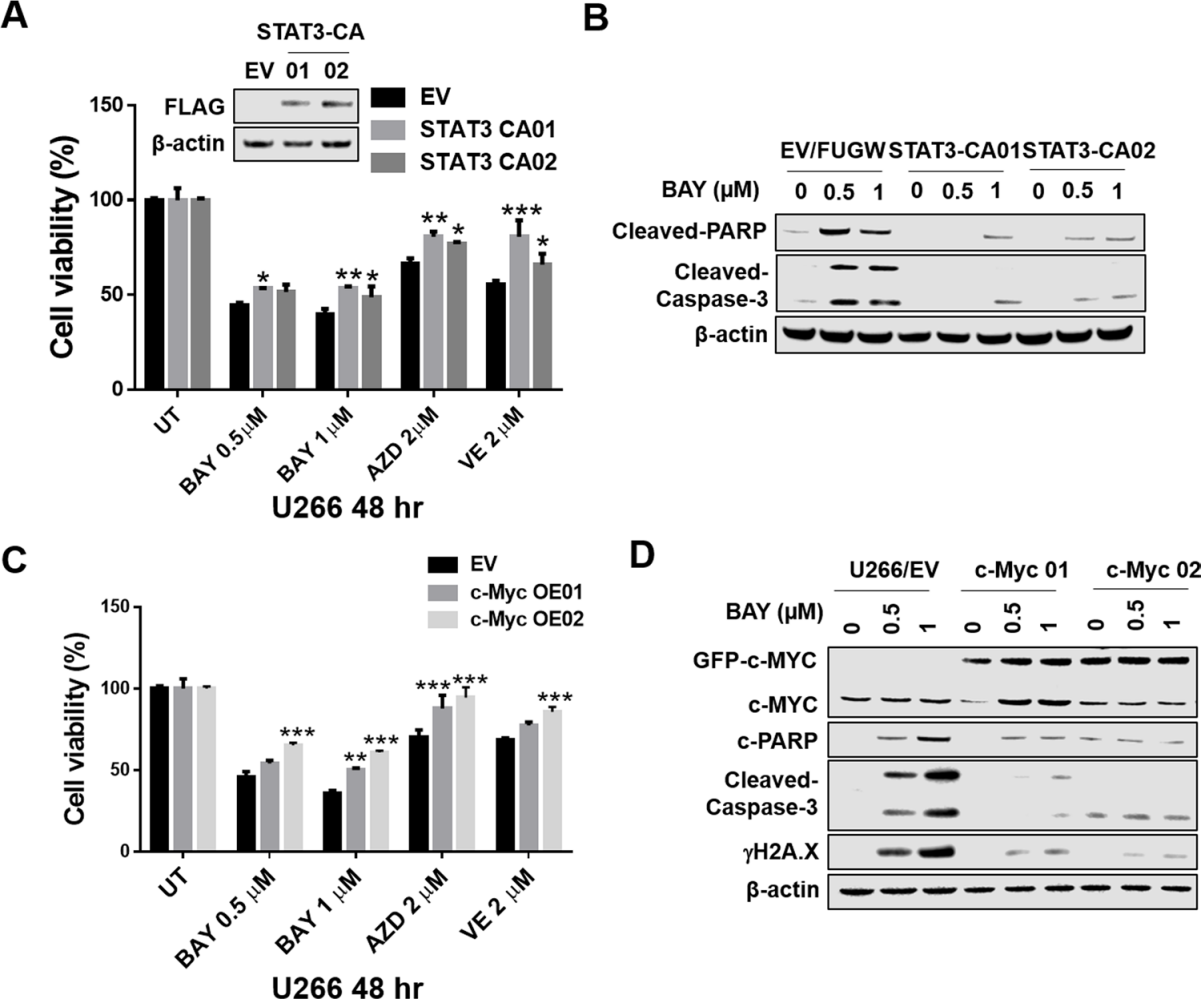
STAT3 inhibition and down-regulation of STAT3 targets by ATR inhibitors play a functional role in lethality. (**A**-**B**) U266 cells were infected with a lentivirus harboring STAT3 CA (FLAG fusion). (**A**) Cells were exposed (48 h) to indicated concentrations of ATR inhibitors, followed by analysis by CellTiter-Glo® Luminescent Cell Viability Assay to monitor cell viability. Values represent the means ± S.D. for three experiments performed in triplicate. * = *P* < 0.05; ** = *P* < 0.01; *** = *P* < 0.001 versus empty-vector control. (**B**) Western blot analysis of cleaved-Caspase-3, and cleaved-PARP in empty-vector and CA-STAT-expressing cells was performed. β-actin controls were assayed to ensure equivalent loading and transfer. (**C**-**D**) U266 cells were transfected with c-Myc overexpressing constructs. (**C**) Cells were exposed (48 h) to the indicated ATR inhibitor concentrations, followed by analysis by the CellTiter-Glo® Luminescent Cell Viability Assay to monitor cell viability. Values represent the means ± S.D. for three experiments performed in triplicate. ** = *P* < 0.01; *** = *P* < 0.001 for c-MYC clones versus empty-vector controls. (**D**) Western blot analysis of c-MYC, cleaved-Caspase-3, cleaved-PARP and γH2A.X was performed in empty-vector and c-MYC-expressing U266 cells. β-actin controls were assayed to ensure equivalent loading and transfer

To investigate the functional significance of STAT3 inactivation on the survival of MM cells exposed to ATR inhibitors, U266 cells were transfected with a vector expressing constitutively active STAT3 (FLAG-labeled CA-STAT3) [21], and subsequently exposed (48 h) to ATR inhibitors (BAY, AZD, VE), after which viability was determined by the Cell-Titer Glo bioluminescence assay. As shown in Fig. 5A, two clones expressing CA-STAT3 (STAT3 CA01 and CA02) displayed a modest but statistically significant increases in survival compared to empty-vector cells following exposure to each of the three ATR inhibitors. They also exhibited concordant reductions in PARP and caspase-3 cleavage (Fig. 5B). Parallel studies performed in cells ectopically expressing the STAT3 downstream target c-MYC (c-MYC 01 and 02) demonstrated a significant increase in survival of over-expressing cells compared to empty-vector controls following exposure to each agent (Fig. 5C). Compatible effects were observed when capase-3 and PARP cleavage as well as γH2A.X were monitored (Fig. 5D). Parallel studies were performed in cells ectopically expressing the STAT3 targets MCL-1 and BCL-X_L_. Enforced expression of MCL-1 significantly reduced the lethal effects of BAY (1 and 2 μM; 48 h; Supplemental Fig. S6A) and diminished PARP and caspase-3 cleavage as well as γH2A.X expression (Supplemental Fig. S6B). Analogous results were observed in cells ectopically expressing BCL-X_L_ (Supplemental Fig. S6C-D). Collectively, these findings argue that inactivation of STAT3 and down-regulation of downstream targets e.g., c-MYC, BCL-X_L_, MCL-1 contribute functionally to cell death induction by ATR inhibitors in MM cells.

### ATR inhibitors diminish p-Y705 expression in primary MM cells

**Fig. 6:**
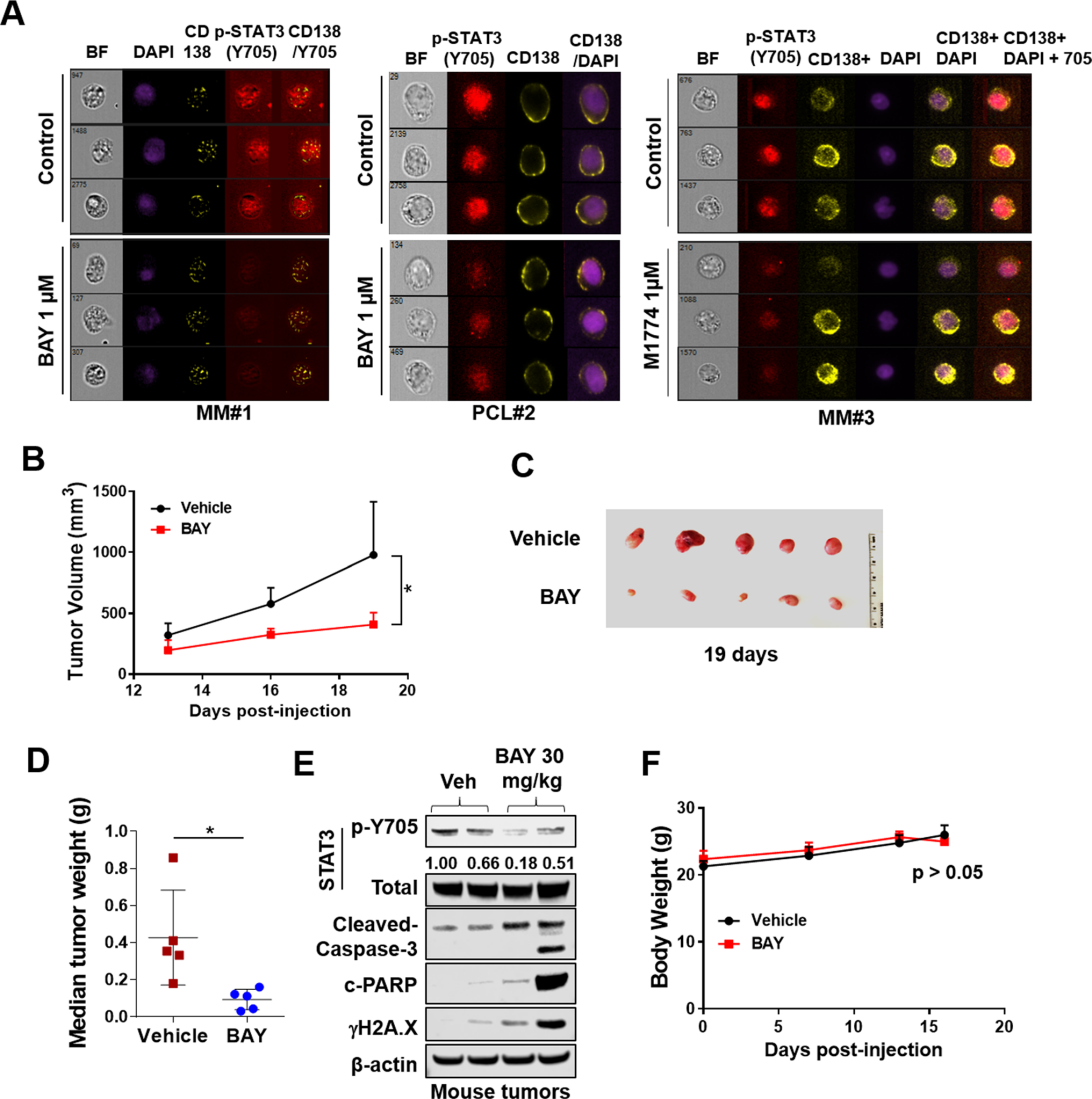
ATR inhibitors decrease p-Y705 STAT3 expression in primary human CD138^+^ MM cells *ex-vivo* and in mice in vivo. (**A**) Isolated primary MM and PCL (plasma cell leukemia) cells from 3 patients were treated with Bay1895344 1 µM or M1774 1 µM for 24 or 20 h, subsequently stained for p-Y705 STAT3, CD138 and DAPI, and visualized by ImageStream. Representative cells are shown (BF = brightfield). (**B**-**F**) NOD/SCID-gamma mice were injected subcutaneously with 5 × 10.^6^ U266 cells in the flank. When tumors grew to 8–10 mm, Bay1895344 (30 mg/kg, p.o., b.i.d.) was administered for 3 days. Controls received equal volumes of vehicle. Tumor growth (**B**) was monitored every other day. Isolated tumors were photographed after 19 days of treatment (**C**). (**D**) Median tumor weight as determined and comparison made between vehicle and BAY1895344. * = P < 0.05. (**E**) Western blot analysis was performed to monitor the indicated candidate pharmacodynamic markers in tumors excised from representative mice. Images were quantified and analyzed by using ImageJ software. (**F**) Mouse body weight was determined at the indicated interval during treatment. There was no significant difference between the treatment groups (P > 0.05)

To determine whether ATRi-mediated STAT3 Y705 phosphorylation could be extrapolated to primary MM cells, CD138^+^ mononuclear cells from bone marrow aspirates obtained from MM patients were assayed by ImageStream analysis for p-STAT3 Y705 expression. As shown for 3 separate MM specimens in Fig. 6A (upper panels), as well as for 2 additional primary MM specimens shown in Supplemental Fig. S7, cells cultured (24 h) in the presence of BAY (1 μM) displayed very clear reductions in p-Y705 expression. Similar results were seen in CD138^+^ cells cultured in the presence of M1774 (Fig. 6A, right panel). Such findings argue that ATR inhibitors disrupt STAT3 pY705 activation in primary MM cells.

### *ATR inhibitors block STAT3 Y705 phosphorylation in an *in vivo* xenograft model in association with MM cell growth inhibition*

To determine whether the preceding findings could be recapitulated in vivo, a U266 xenograft model was employed. NSG mice (5 mice/group) were injected in the flank with 5 × 10^6^ MM cells and treated with vehicle or BAY 30 mg/kg for 3 days. At varying periods beyond 12 days, tumor size was monitored by caliper measurement. As shown in Fig. 6B-C), tumor size was significantly reduced in the treatment group compared to saline controls (P < 0.05). Alternatively, mice were sacrificed on d19, and tumors excised and weighed. A significant reduction in tumor weights was observed in treated animals (P < 0.05; Fig. 6D). Moreover, western blot analysis of proteins extracted from excised tumor revealed modest but discernable reductions in STAT3 p-Y705 expression compared to controls, as well as an increase in Caspase-3 cleavage, PARP cleavage and γH2A.X expression (Fig. 6E). This treatment regimen was not associated with significant weight loss (Fig. 6F). These findings indicate that the ability of ATR inhibitors to disrupt STAT3 Y705 phosphorylation in MM cells as observed in vitro can be recapitulated in the in vivo setting.

## Discussion

The STAT3 transcription factor plays a critical role in the survival and proliferation of diverse tumor cell types, particularly MM [22]. It has also been implicated in the development of resistance to anti-cancer agents, including that mediated by micro-environmental factors [23]. The present studies indicate that in MM cells, disruption of ATR signaling opposes activation of STAT3, a phenomenon that is likely to involve diminished phosphorylation of STAT3 Y705, an event necessary for STAT3 dimerization and subsequent nuclear translocation [24]. In this context, disruption of ATR recapitulates, at least in part, the consequences of inhibition of its downstream target, Chk1, which we have recently shown to be directly responsible for phosphorylation of STAT3 at this site [15]. Consistent with these observations, ATR inhibition, like that of Chk1 inhibition, failed to diminish STAT3 S727 phosphorylation, responsible for STAT3 DNA binding and activation in malignant B-cells [25]. Despite these findings, we cannot presently rule out the possibility that ATR inhibitor off-target effects may also contribute to the observed inactivation of STAT3. In any event, the significance of these findings stems from the fact that for multiple reasons (e.g., toxicity), the clinical development of Chk1 inhibitors has largely been paused, whereas ATR inhibitor development continues unabated [6]. The ability of ATR inhibitors to disrupt STAT3 function, critically important in MM cell survival, along with existing evidence that such cells are highly vulnerable to ATR inhibitor-induced replicative stress [7], makes development of these agents for a disease such as MM particularly attractive.

Very little information currently exists concerning the relationship between STAT3 and ATR signaling, and almost all of this involves examination of the effects of STAT3 on ATR activation. Furthermore, much of this information is cell context-specific and to a certain extent contradictory. For example, in fibrosarcoma and CHO cells, diminished STAT3 activity was associated with reduced ATR induction [26]. Conversely, STAT3 was shown to interrupt ATR/Chk1 signaling in EBV-infected lymphocytes [27] and in Marek’s disease-infected fibroblasts, STAT3 inactivated the ATR/Chk1 pathway [28]. In contrast, results of the present study argue that ATR plays a key role in maintaining STAT3 activation in MM cells, and that interrupting ATR signaling represents a potentially effective strategy to disable STAT3 and antagonize its cytoprotective actions. Given evidence of the dependence of MM cells to STAT3 activation for survival [29], it is tempting to speculate that this approach may exert disparate effects on normal versus neoplastic cells. Further studies will be necessary to confirm or refute this notion.

Importantly, the ability of ATR inhibitors to inactivate STAT3 at Y705 was recapitulated by ATR siRNA knock-down, arguing for an on-target mechanism of action. It was also accompanied by down-regulation of multiple STAT3 downstream cytoprotective targets e.g., MCL-1, BCL-X_L_, and c-Myc. Of these, MCL-1 and c-Myc are known to play particularly important roles in MM cell survival [30, 31]. Moreover, expression of MCL-1 and BCL-X_L_ are both tightly regulated by IL-6 [32] The ability of ectopic expression of these proteins to protect MM cells, albeit partially, from ATR inhibitor-mediated cell death argues that down-regulation of these proteins plays a significant role in anti-MM actions. Analogously, the capacity of constitutively active STAT3 to protect cells from ATR inhibitor lethality supports the notion that STAT3 inactivation contributes functionally to ATRi-mediated activity. Finally, it is noteworthy that similar events were also observed in MM cells highly resistant to the proteasome inhibitor bortezomib [16], as well as in IL-6-stimulated cells (OPM2) cultured in the presence of this cytokine or stromal cell conditioned medium. In this context, IL-6 has been strongly implicated in the development of microenvironmental forms of resistance through a STAT3-dependent mechanism [33]. These observations raise the possibility that ATR inhibitor-based strategies may be effective against cells resistant to various other anti-MM agents through such mechanisms.

The ability of ATR inhibitors to block STAT3 Y705 phosphorylation was recapitulated in primary CD138^+^ cells as well as in MM cells extracted from NSG mice and treated in vivo with BAY. Notably, the latter findings occurred with ATR inhibitor doses that effectively reduced tumor burden and were unassociated with significant toxicity. The in vivo activity of ATR inhibitors in the present study is consistent with evidence that MM cells may be particularly vulnerable to replication stress [7, 34], which represents an important mediator of ATR inhibitor-related actions [35]. The significance of the present findings is that in light of evidence that STAT3 plays an important role in protecting tumor cells from the lethal consequences of DNA damage e.g., oxidative stress [26, 36], it is plausible that disabling STAT3 may represent an additional mechanism by which ATR inhibitors trigger cell death in MM cells. Aside from the possibility that STAT3 Y705 could represent a candidate pharmacodynamic biomarker of ATR inhibitor activity in this disease, it is conceivable that MM cells with high basal STAT3 activity, and presumably dependent upon this transcription factor for survival, may be particularly susceptible to ATR inhibitor-based strategies. Accordingly, attempts to test this biomarker hypothesis are currently underway.

### Supplementary Information

Below is the link to the electronic supplementary material.

Fig. S1 ATR inhibitors induce apoptosis in drug-resistant multiple myeloma cells. (A-C) Bortezomib-resistant PS-R cells were exposed (24 hr) to 3 nM or 30 nM bortezomib (BTZ) or the indicated concentrations of Bay1895344 or AZD6738 (48 hr) followed by flow cytometric analysis to monitor the percentage of apoptotic (7-AAD+) cells. Values represent the means ± S.D. for three experiments performed in triplicate. * = P < 0.05; ** = P < 0.01; *** = P < 0.001 = significantly greater than values for untreated controls. (D-E) PS-R cells were incubated with the indicated concentrations of Bay1895344 or M1774 for 24 hours, after which γH2A.X and cleavage of caspase-3, or cleavage of PARP were monitored by immunoblotting analysis. β-actin was assayed to ensure equivalent loading and transfer. Results are representative of 3 separate experiments (PNG 97 kb)

High resolution image (TIF 192 KB)

Fig. S2 ATR inhibitors induce apoptosis in multiple myeloma cells. H929, KMS11, RPMI8226, RPMI8226/V10R, and KAS/6-1 cells (± 1 ng/mL IL-6) were exposed to the indicated concentrations of Bay1895344 or M1774 for 24 or 48 hours, followed by flow cytometric analysis of cell death after staining with 7-AAD. Values represent the means ± S.D. for three experiments performed in triplicate. * = P < 0.05; ** = P < 0.01; *** = P < 0.001 = significantly greater than values for untreated controls. Results are representative of 3 separate experiments (PNG 89 kb)

High resolution image (TIF 225 KB)

Fig. S3 ATR inhibitors block tyrosine phosphorylation of STAT3 (p-Y705) and inhibit STAT3 signaling pathways, as well as induce apoptosis in MM cells. Western blot analysis of p-Y705 STAT3, p-S727 STAT3, total STAT3, γH2A.X and cleavage of caspase-3, or cleavage of PARP in RPMI8226, RPMI8226/V10R and KAS/6-1 cells treated with indicated concentrations of Bay1895344, or M1774 for 24 hours. β-actin control was assayed to ensure equivalent loading and transfer (PNG 660 kb)

High resolution image (TIF 276 KB)

Fig. S4 ATR inhibitors block tyrosine phosphorylation of STAT3 (p-Y705) and inhibit STAT3 signaling pathway in MM cells. (A-B) Western blot analysis of p-Y705 STAT3, p-S727 STAT3, total STAT3 and the STAT3 downstream targets MCL-1, BCL-XL, c-Myc, in PSR cells treated with indicated concentrations of Bay1895344 and M1774 for 24 hours. α-tubulin, β-actin or GAPDH controls were assayed to ensure equivalent loading and transfer. (C-D) OPM2 cells were pretreated with patient-derived stromal cell-conditioned medium (PDCM, 6 hr). WB analysis of p- or total-STAT3, and c-MYC was performed in OPM2 cells exposed to Bay1895344 or M1774 for 24 hours. Images were quantified by densitometry and analyzed using ImageJ software (PNG 159 kb)

High resolution image (TIF 306 KB)

Fig. S5 ATR inhibitors exert modest effects on the expression level of SOCS1/3 in MM cells. (A-D) Western blot analysis of SOCS3 in U266 and PSR cells treated with indicated concentrations of Bay1895344 and M1774 for 16 or 24 hours. (E-G) Western blot analysis of SOCS1/3 in RPMI8226, RPMI8226/V10R and KAS/6-1 cells treated with indicated concentrations of Bay1895344 for 24 hours. β-actin control was assayed to ensure equivalent loading and transfer (PNG 120 kb)

High resolution image (TIF 231 KB)

Fig. S6 Down-regulation of the STAT3 targets MCL-1 and BCL-XL by ATR inhibition plays a functional role in ATR inhibitor lethality. (A-B) U266 cells were transfected with MCL-1, overexpressing constructs, after which they were exposed (48 hr) to indicated concentrations of BAY, followed by the CellTiter-Glo® Luminescent Cell Viability Assay to monitor cell viability. (B) Western blot analysis of MCL-1, cleaved-Caspase-3, cleaved-PARP, and γH2A.X was performed in empty-vector and MCL-1-overexpressing cells. β-actin controls were assayed to ensure equivalent loading and transfer. (C-D) U266 cells were infected with a lentivirus harboring BCL-XL. (C) Cells were exposed (48 hr) to indicated concentrations of BAY, followed by the CellTiter-Glo® Luminescent Cell Viability Assay to monitor cell viability. (D) Western blot analysis of BCL-X_L_, cleaved-Caspase-3, cleaved-PARP, and γH2A.X was performed. β-actin controls were assayed to ensure equivalent loading and transfer. Values represent the means ± S.D. for three experiments performed in triplicate. * = P < 0.05; ** = P < 0.01 for values for empty-vector controls versus cells ectopically expressing MCL-1 or BCL-X_L_ (PNG 104 kb)

High resolution image (TIF 192 KB)

Fig. S7 ATR inhibitor decreases p-Y705 STAT3 expression in primary human CD138^+^ MM cells *ex-vivo*. Isolated primary MM cells from two additional MM patients were treated with Bay1895344 1 µM for 24 hr, and subsequently stained with DAPI and with antibodies to p-Y705, S727 and CD138, after which they were visualized by ImageStream. Results for representative cells are shown. (BF = brightfield) (PNG 453 kb)

High resolution image (TIF 598 KB)

Supplementary file8 (XLSX 17 KB)

## Data Availability

The datasets generated during and/or analysed during the current study are available from the corresponding author on reasonable request. We would like to note that all updated original source data (chiefly Western blot data) is. available to the journal and readers via storage on the website OSFHOME with the following web address: https://osf.io/68kuq/?view_only=4bbc9c6bc0fa4c2bbf8c9b6a6608ad2b.
