# Supplementary figures and images for: Non-canonical role for the ataxia-telangiectasia-Rad3 pathway in STAT3 activation in human multiple myeloma cells
Source: Cell Oncol (Dordr). 2023 May 1;46(5):1369–80. doi: 10.1007/s13402-023-00817-6 (PMC10618375; doi:10.1007/s13402-023-00817-6)

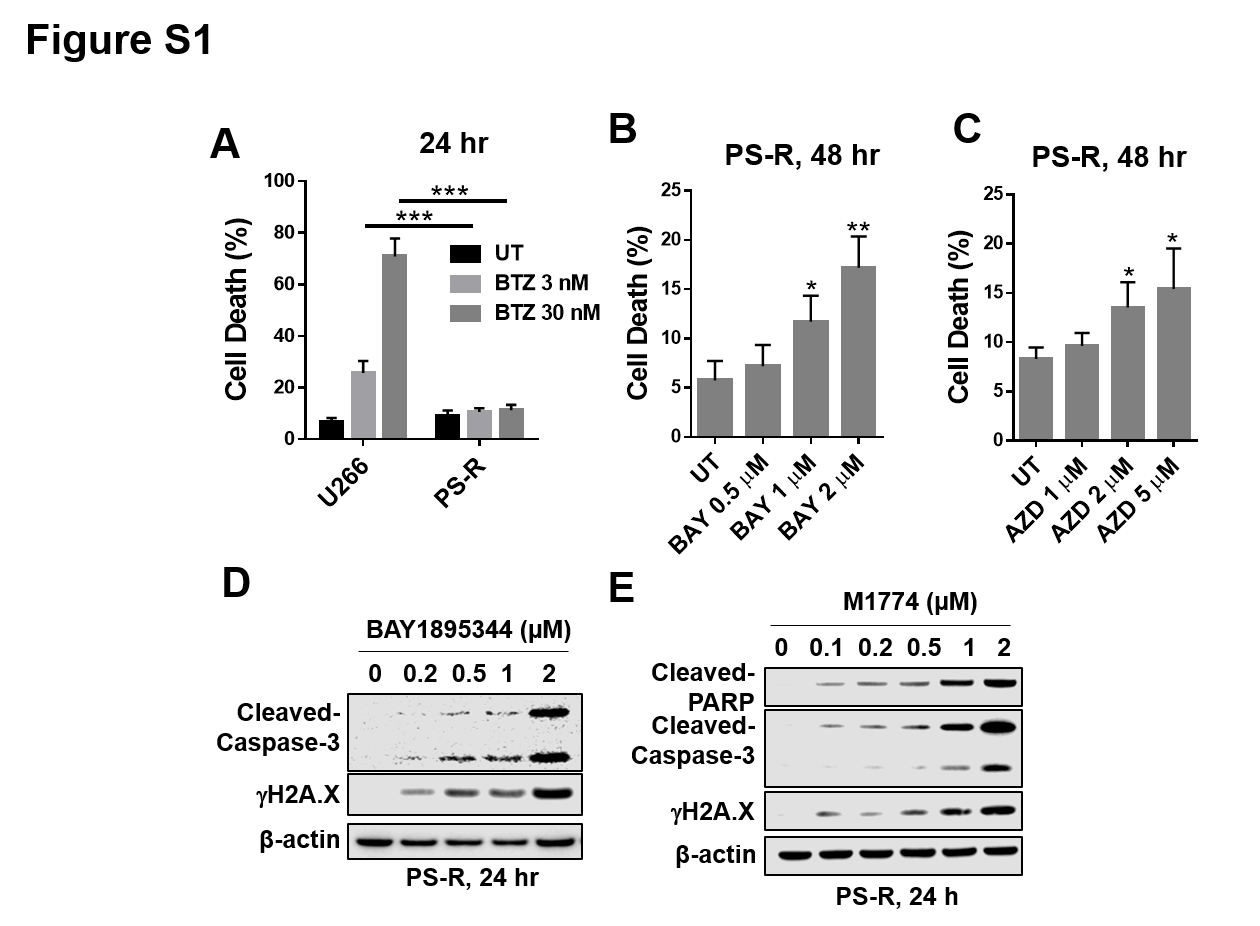

Supplement: Supplementary file 1 — ATR inhibitors induce apoptosis in drug-resistant multiple myeloma cells. (A-C) Bortezomib-resistant PS-R cells were exposed (24 hr) to 3 nM or 30 nM bortezomib (BTZ) or the indicated concentrations of Bay1895344 or AZD6738 (48 hr) followed by flow cytometric analysis to monitor the percentage of apoptotic (7-AAD+) cells. Values represent the means ± S.D. for three experiments performed in triplicate. * = P < 0.05; ** = P < 0.01; *** = P < 0.001 = significantly greater than values for untreated controls. (D-E) PS-R cells were incubated with the indicated concentrations of Bay1895344 or M1774 for 24 hours, after which γH2A.X and cleavage of caspase-3, or cleavage of PARP were monitored by immunoblotting analysis. β-actin was assayed to ensure equivalent loading and transfer. Results are representative of 3 separate experiments (PNG 97 kb) [file 13402_2023_817_Fig7_ESM.png]

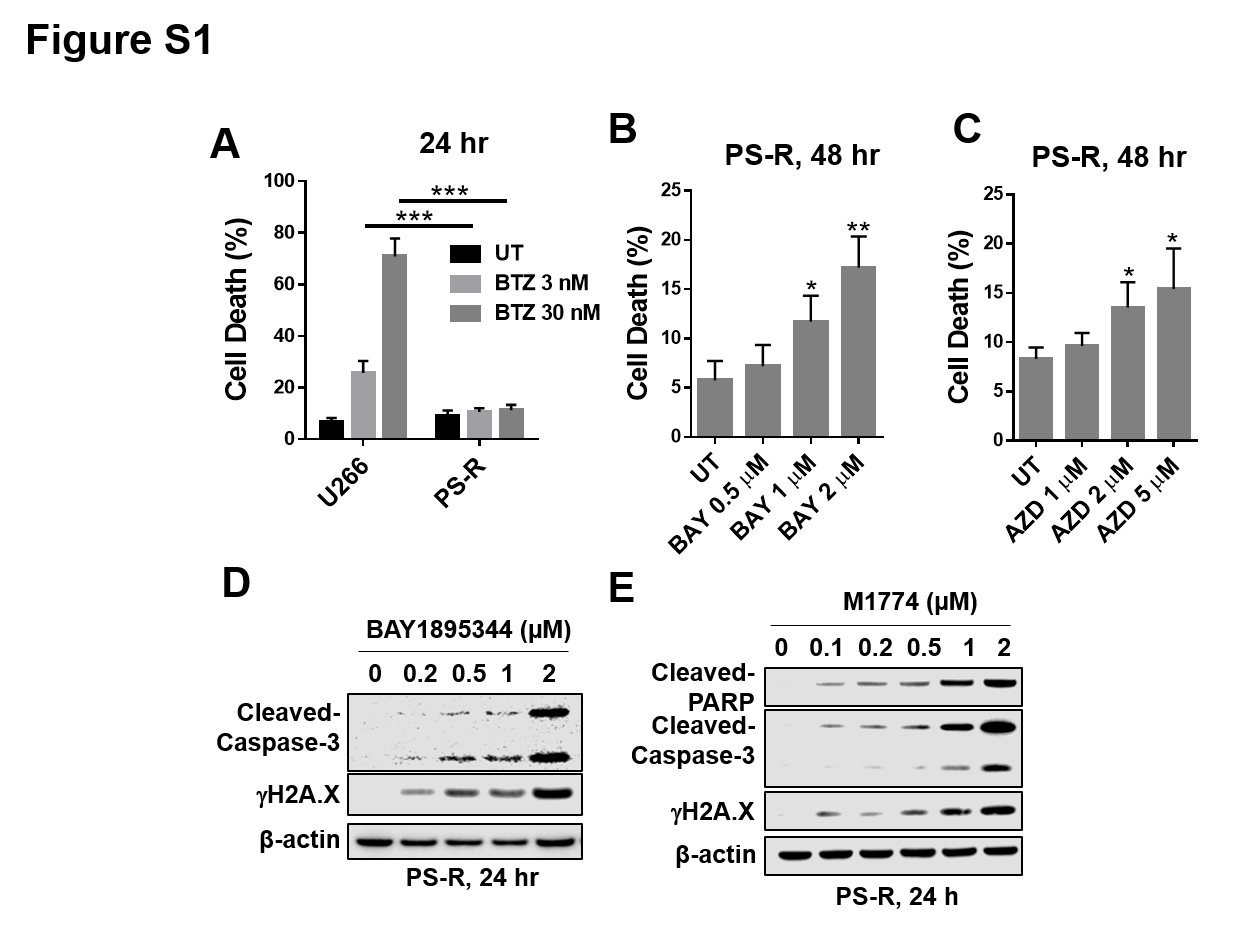

Supplement: Supplementary file 2 — High resolution image (TIF 192 KB) [file 13402_2023_817_MOESM1_ESM.tif]

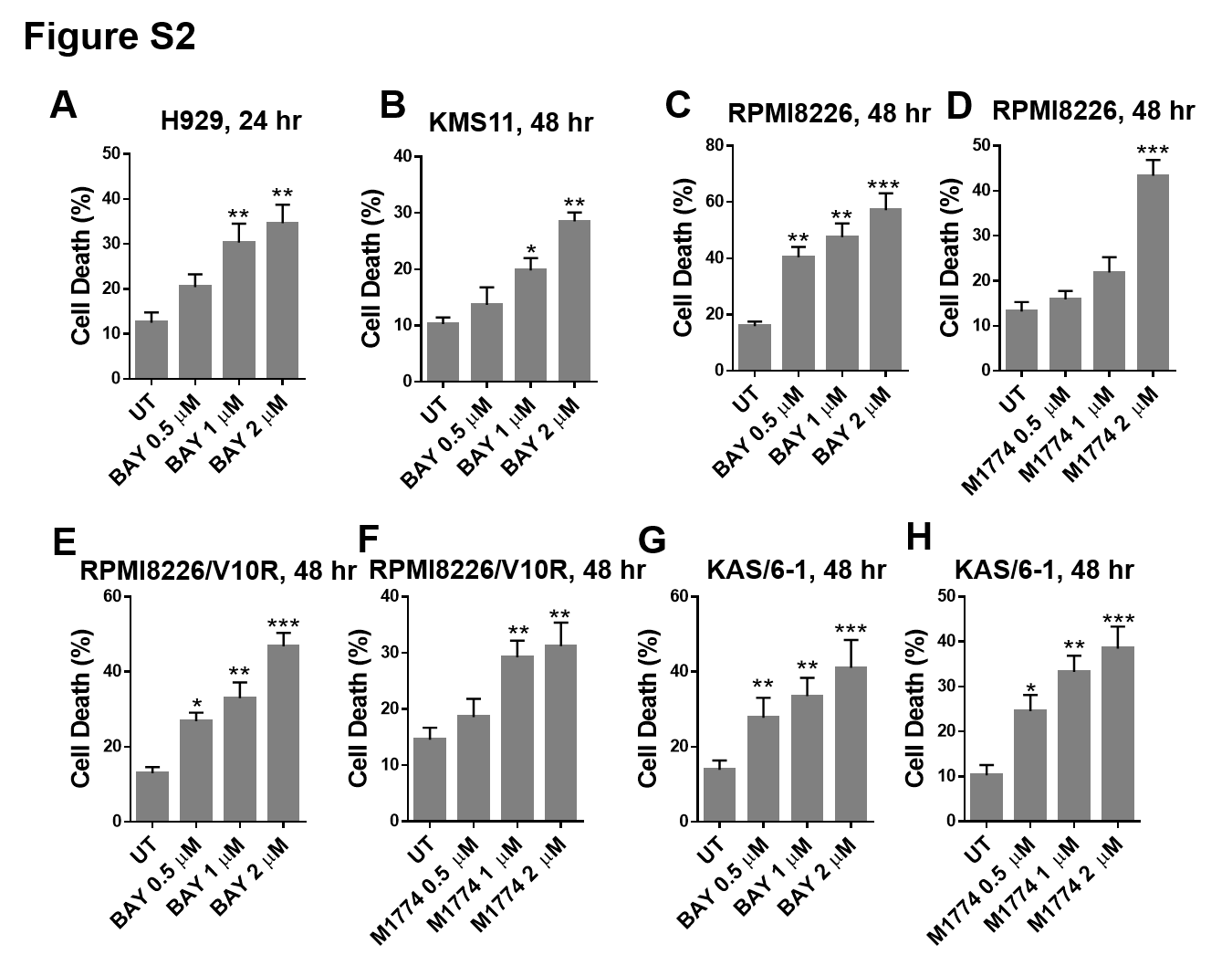

Supplement: Supplementary file 3 — ATR inhibitors induce apoptosis in multiple myeloma cells. H929, KMS11, RPMI8226, RPMI8226/V10R, and KAS/6-1 cells (± 1 ng/mL IL-6) were exposed to the indicated concentrations of Bay1895344 or M1774 for 24 or 48 hours, followed by flow cytometric analysis of cell death after staining with 7-AAD. Values represent the means ± S.D. for three experiments performed in triplicate. * = P < 0.05; ** = P < 0.01; *** = P < 0.001 = significantly greater than values for untreated controls. Results are representative of 3 separate experiments (PNG 89 kb) [file 13402_2023_817_Fig8_ESM.png]

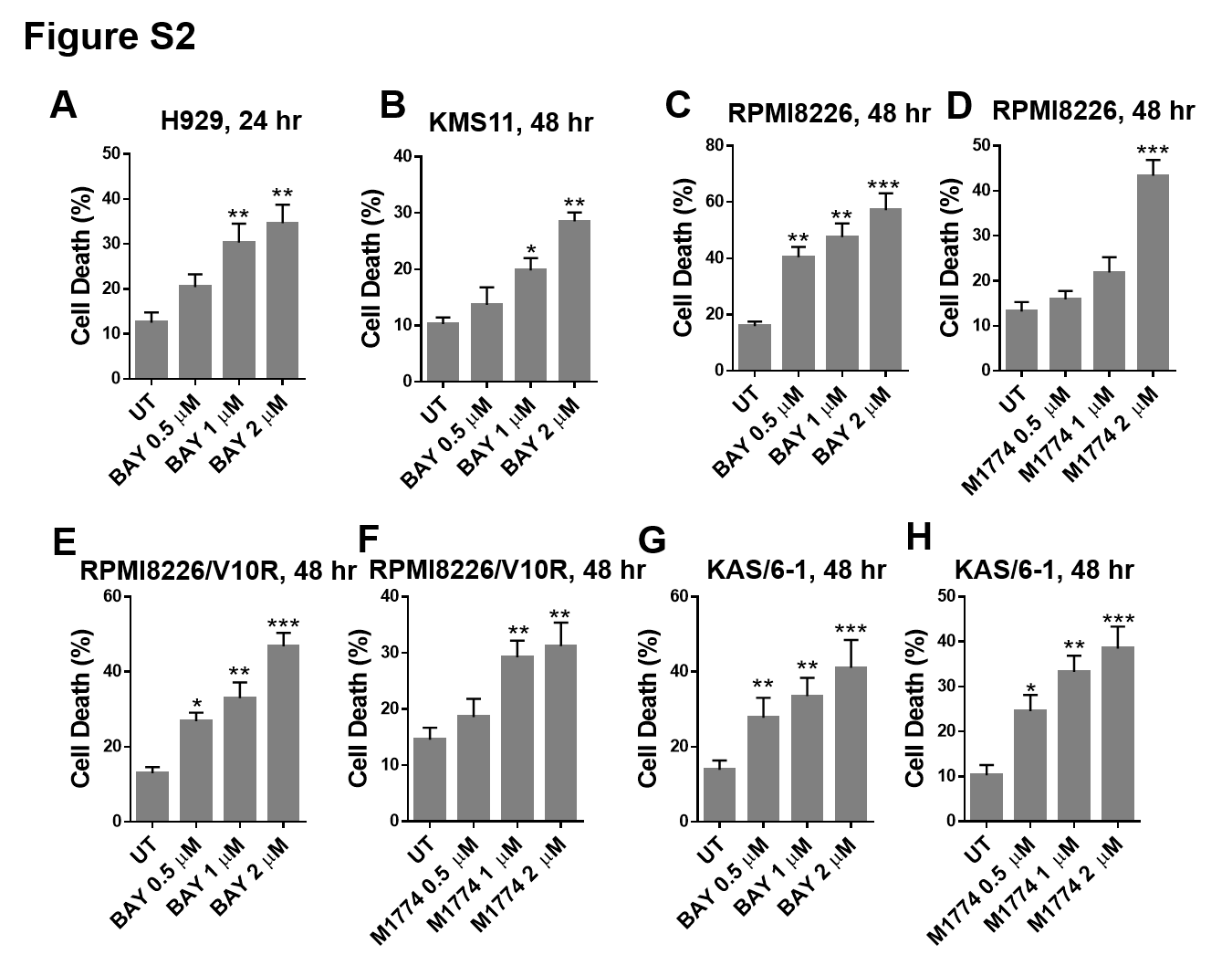

Supplement: Supplementary file 4 — High resolution image (TIF 225 KB) [file 13402_2023_817_MOESM2_ESM.tif]

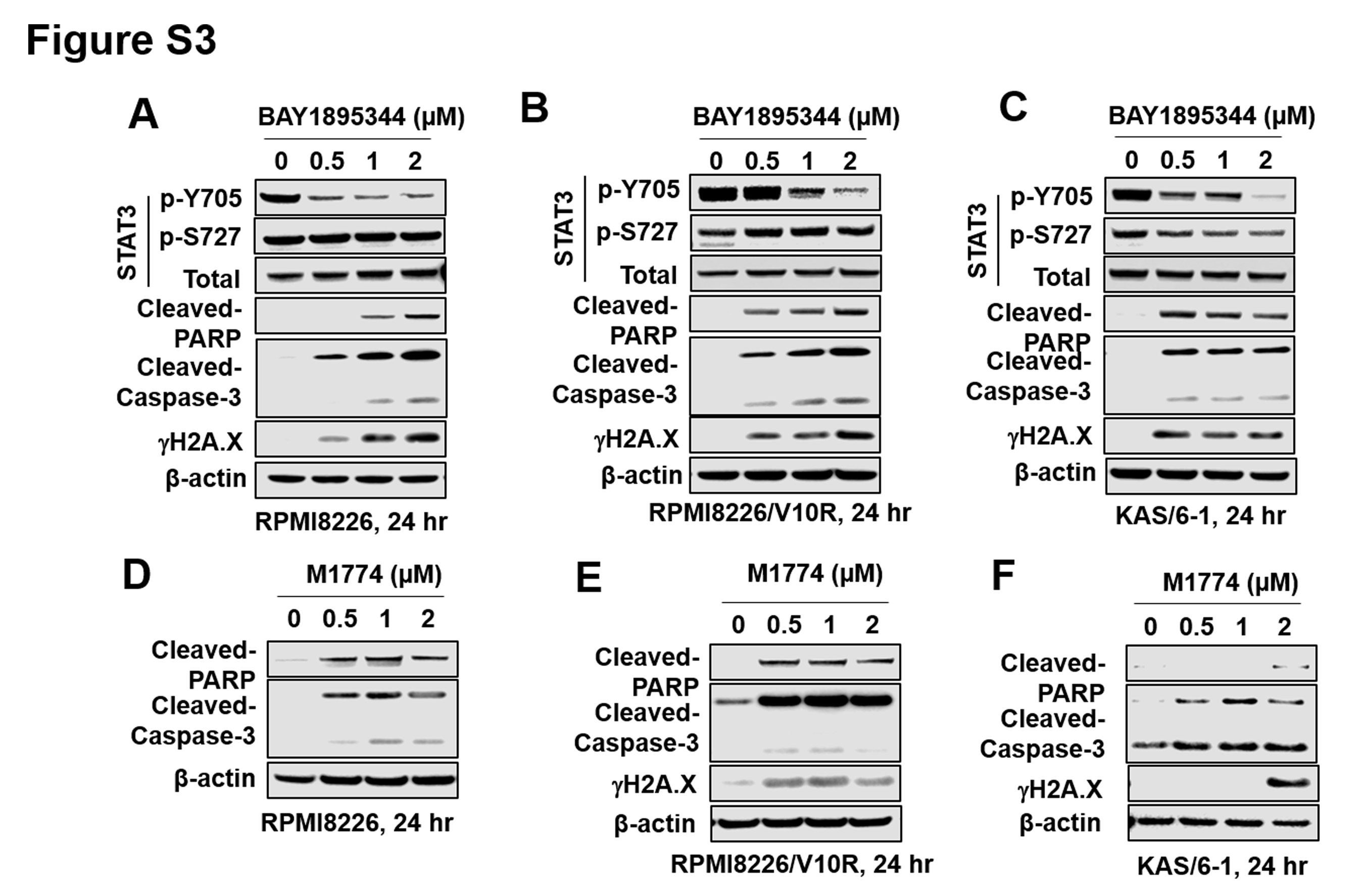

Supplement: Supplementary file 5 — ATR inhibitors block tyrosine phosphorylation of STAT3 (p-Y705) and inhibit STAT3 signaling pathways, as well as induce apoptosis in MM cells. Western blot analysis of p-Y705 STAT3, p-S727 STAT3, total STAT3, γH2A.X and cleavage of caspase-3, or cleavage of PARP in RPMI8226, RPMI8226/V10R and KAS/6-1 cells treated with indicated concentrations of Bay1895344, or M1774 for 24 hours. β-actin control was assayed to ensure equivalent loading and transfer (PNG 660 kb) [file 13402_2023_817_Fig9_ESM.png]

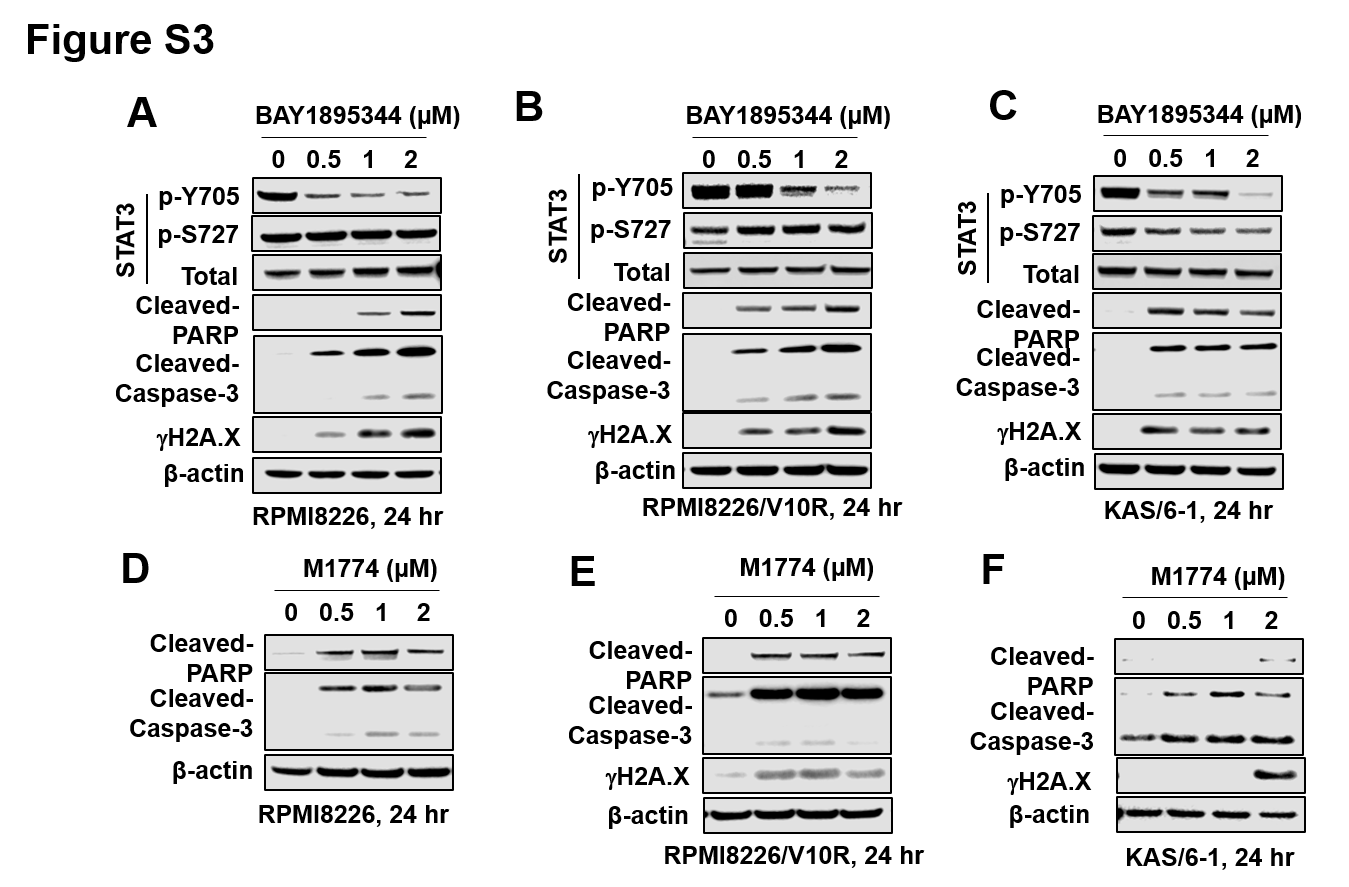

Supplement: Supplementary file 6 — High resolution image (TIF 276 KB) [file 13402_2023_817_MOESM3_ESM.tif]

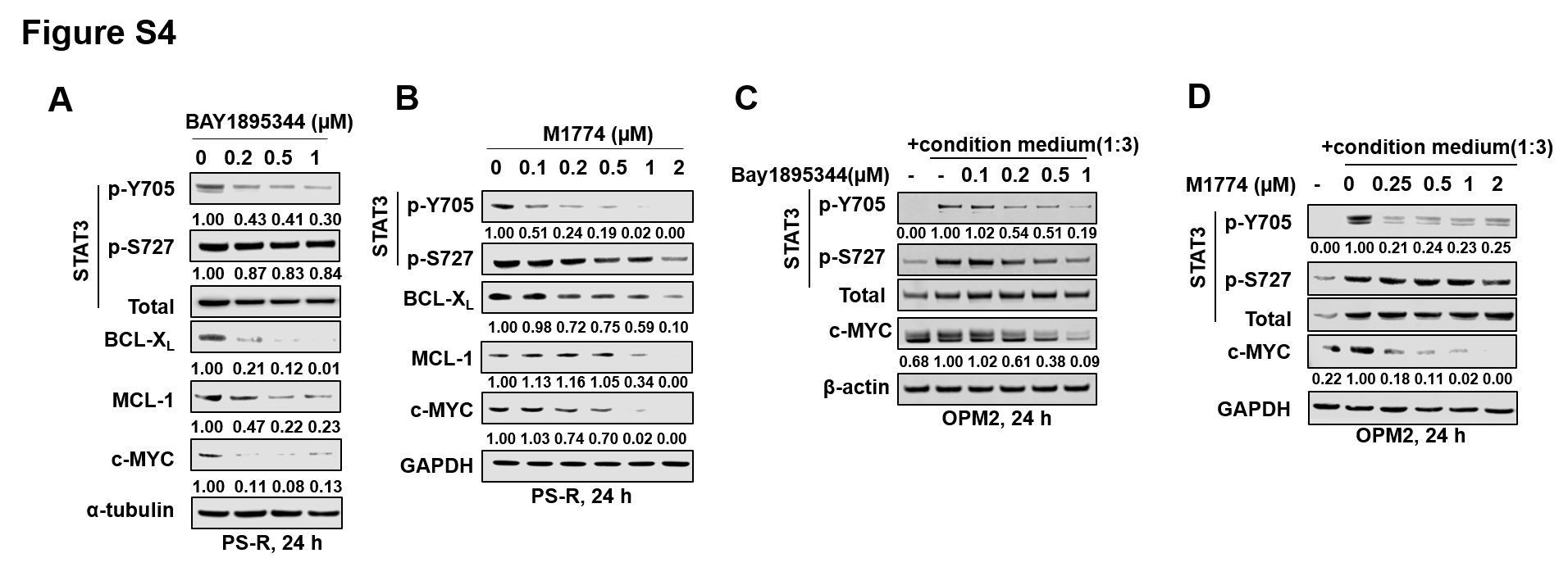

Supplement: Supplementary file 7 — ATR inhibitors block tyrosine phosphorylation of STAT3 (p-Y705) and inhibit STAT3 signaling pathway in MM cells. (A-B) Western blot analysis of p-Y705 STAT3, p-S727 STAT3, total STAT3 and the STAT3 downstream targets MCL-1, BCL-XL, c-Myc, in PSR cells treated with indicated concentrations of Bay1895344 and M1774 for 24 hours. α-tubulin, β-actin or GAPDH controls were assayed to ensure equivalent loading and transfer. (C-D) OPM2 cells were pretreated with patient-derived stromal cell-conditioned medium (PDCM, 6 hr). WB analysis of p- or total-STAT3, and c-MYC was performed in OPM2 cells exposed to Bay1895344 or M1774 for 24 hours. Images were quantified by densitometry and analyzed using ImageJ software (PNG 159 kb) [file 13402_2023_817_Fig10_ESM.png]

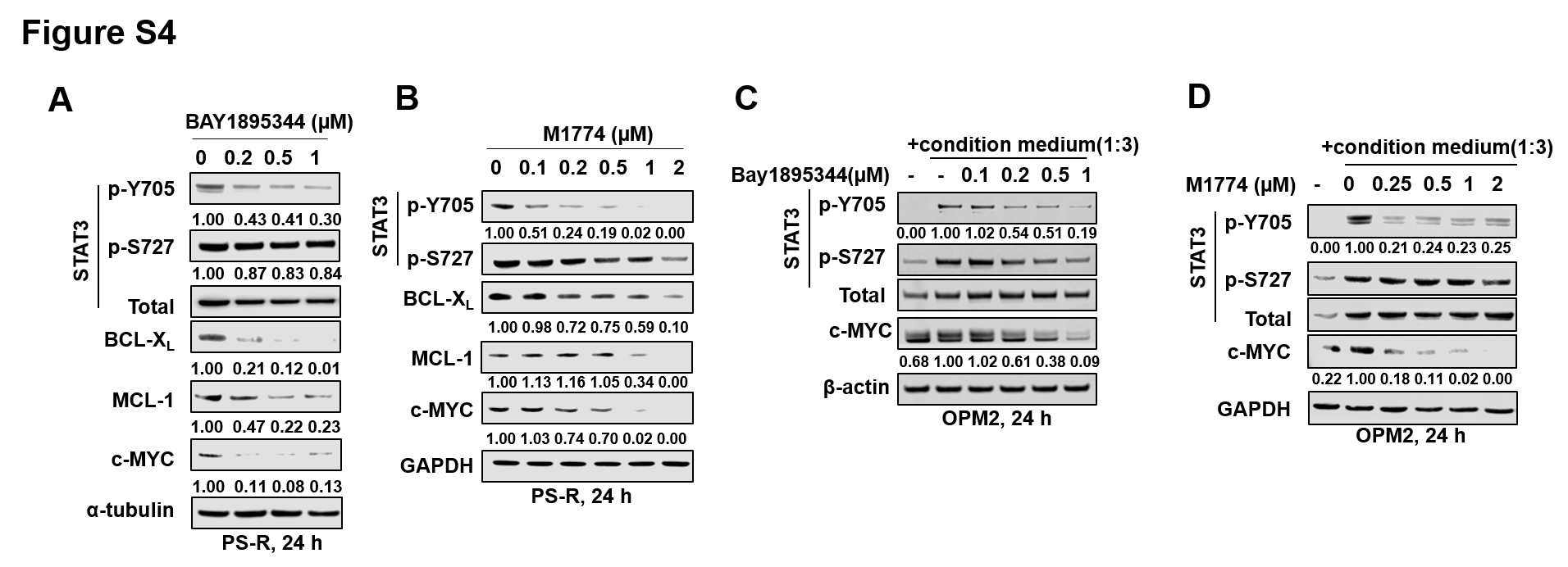

Supplement: Supplementary file 8 — High resolution image (TIF 306 KB) [file 13402_2023_817_MOESM4_ESM.tif]

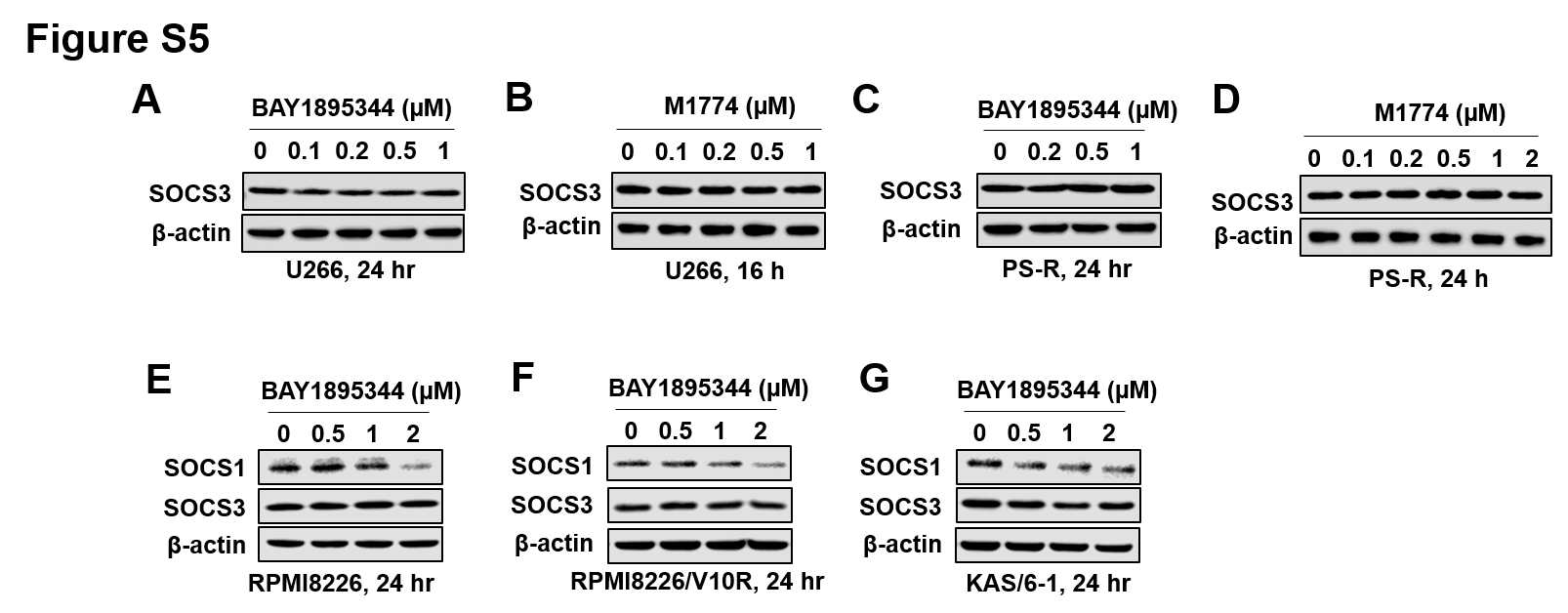

Supplement: Supplementary file 9 — ATR inhibitors exert modest effects on the expression level of SOCS1/3 in MM cells. (A-D) Western blot analysis of SOCS3 in U266 and PSR cells treated with indicated concentrations of Bay1895344 and M1774 for 16 or 24 hours. (E-G) Western blot analysis of SOCS1/3 in RPMI8226, RPMI8226/V10R and KAS/6-1 cells treated with indicated concentrations of Bay1895344 for 24 hours. β-actin control was assayed to ensure equivalent loading and transfer (PNG 120 kb) [file 13402_2023_817_Fig11_ESM.png]

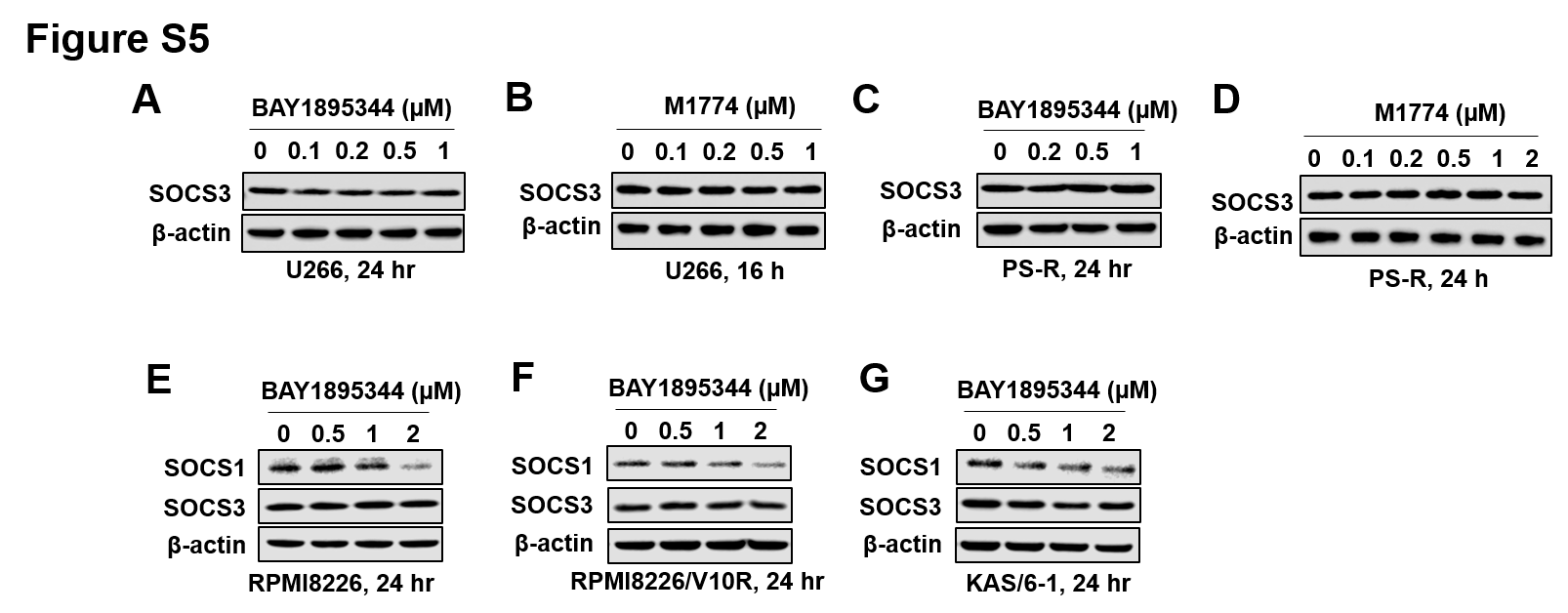

Supplement: Supplementary file 10 — High resolution image (TIF 231 KB) [file 13402_2023_817_MOESM5_ESM.tif]

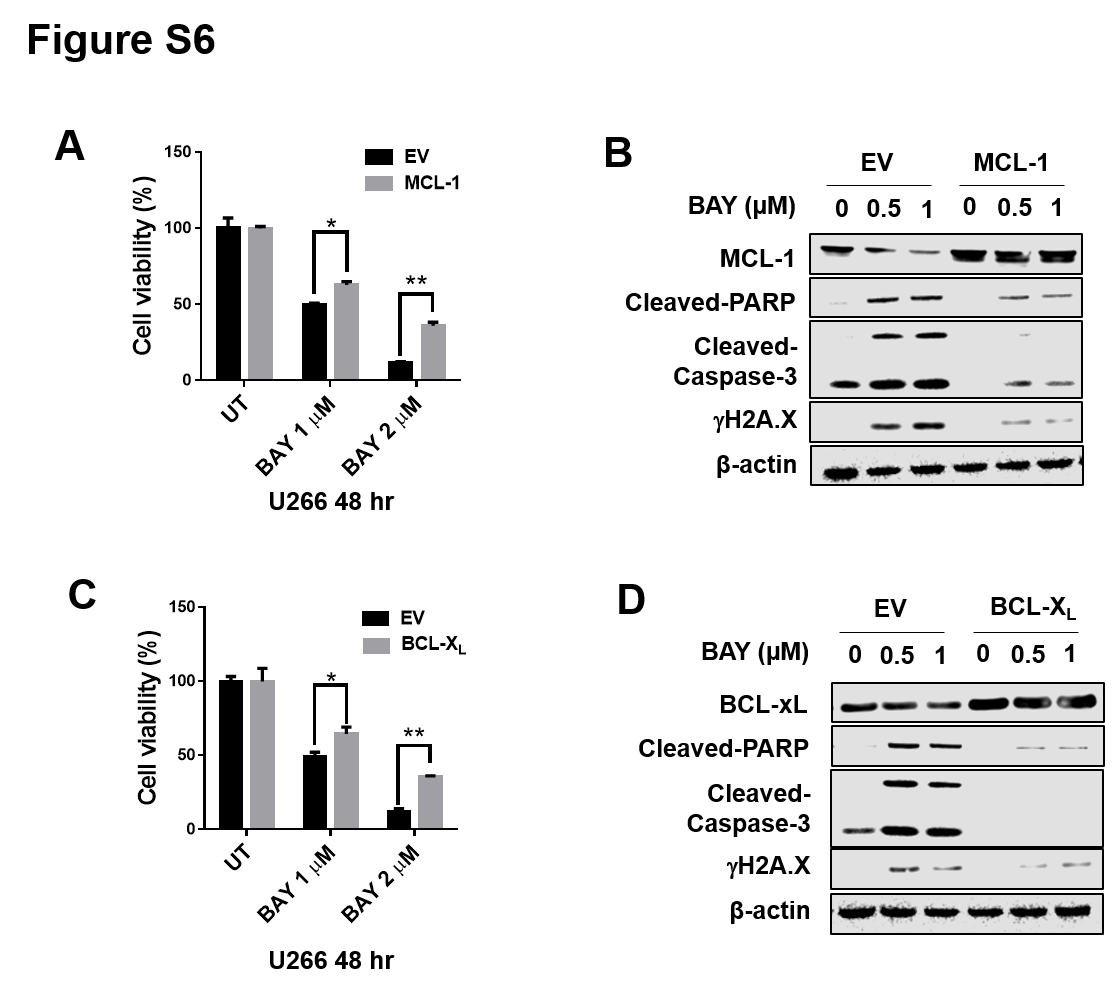

Supplement: Supplementary file 11 — Down-regulation of the STAT3 targets MCL-1 and BCL-XL by ATR inhibition plays a functional role in ATR inhibitor lethality. (A-B) U266 cells were transfected with MCL-1, overexpressing constructs, after which they were exposed (48 hr) to indicated concentrations of BAY, followed by the CellTiter-Glo® Luminescent Cell Viability Assay to monitor cell viability. (B) Western blot analysis of MCL-1, cleaved-Caspase-3, cleaved-PARP, and γH2A.X was performed in empty-vector and MCL-1-overexpressing cells. β-actin controls were assayed to ensure equivalent loading and transfer. (C-D) U266 cells were infected with a lentivirus harboring BCL-XL. (C) Cells were exposed (48 hr) to indicated concentrations of BAY, followed by the CellTiter-Glo® Luminescent Cell Viability Assay to monitor cell viability. (D) Western blot analysis of BCL-XL, cleaved-Caspase-3, cleaved-PARP, and γH2A.X was performed. β-actin controls were assayed to ensure equivalent loading and transfer. Values represent the means ± S.D. for three experiments performed in triplicate. * = P < 0.05; ** = P < 0.01 for values for empty-vector controls versus cells ectopically expressing MCL-1 or BCL-XL (PNG 104 kb) [file 13402_2023_817_Fig12_ESM.png]

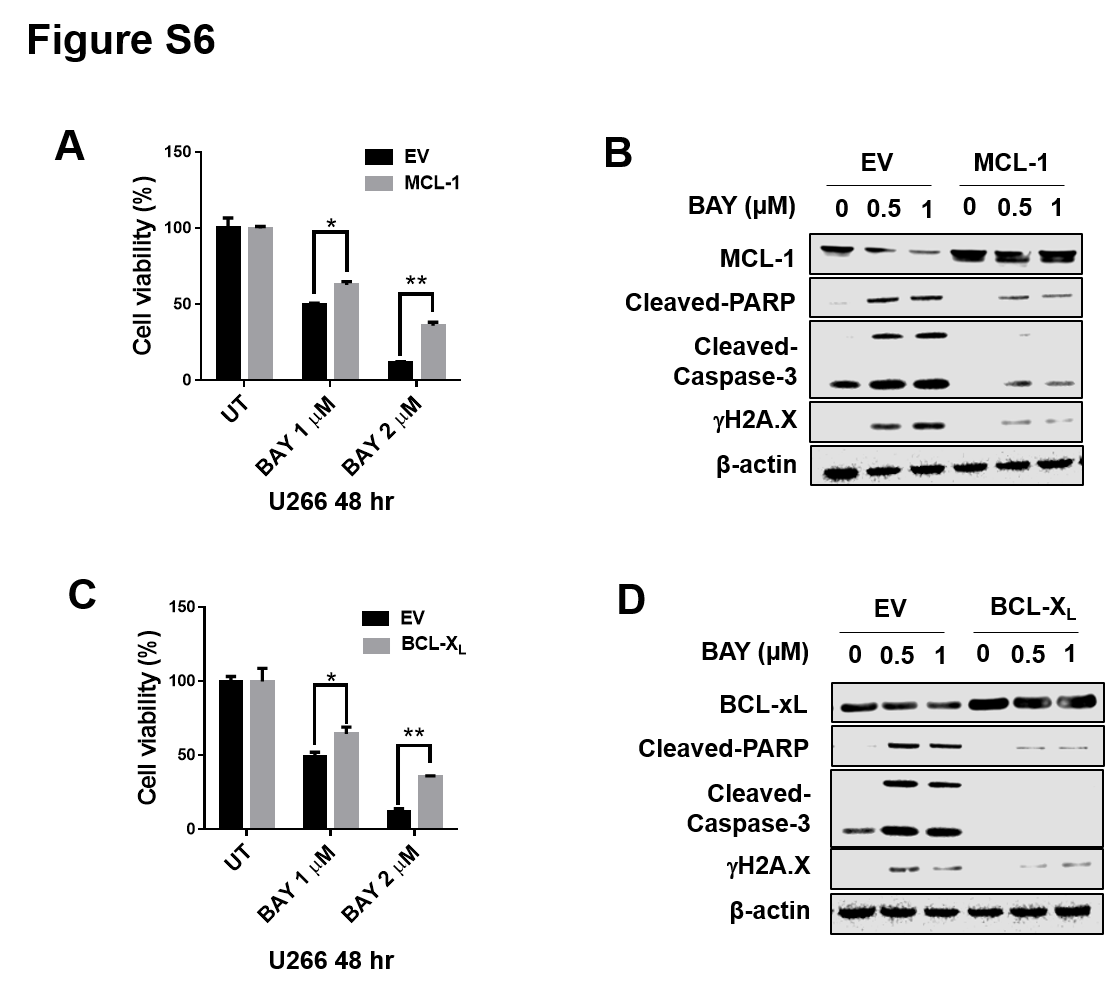

Supplement: Supplementary file 12 — High resolution image (TIF 192 KB) [file 13402_2023_817_MOESM6_ESM.tif]

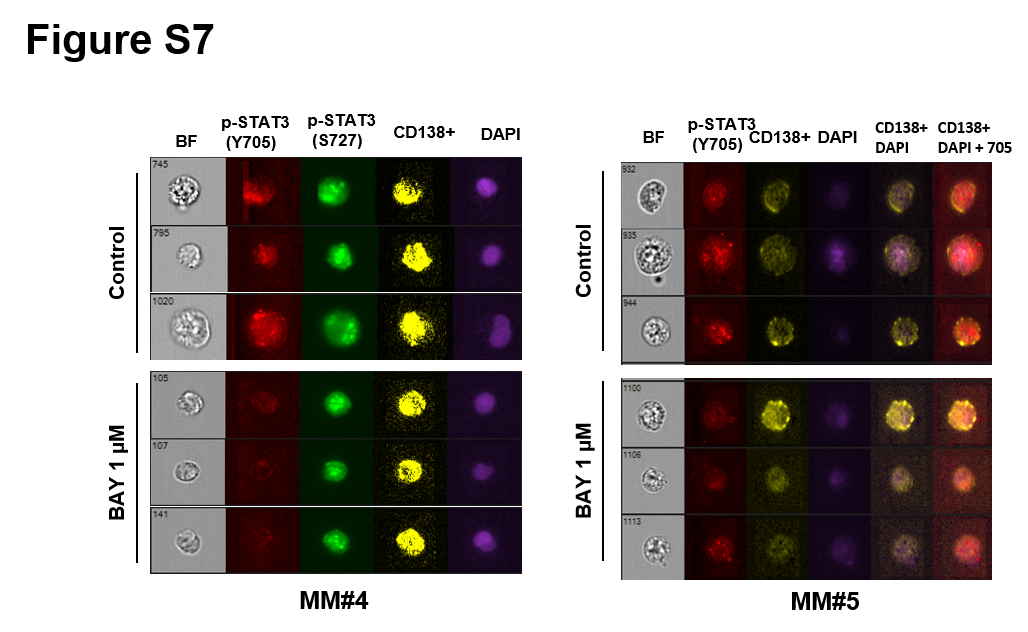

Supplement: Supplementary file 13 — ATR inhibitor decreases p-Y705 STAT3 expression in primary human CD138+ MM cells ex-vivo. Isolated primary MM cells from two additional MM patients were treated with Bay1895344 1 µM for 24 hr, and subsequently stained with DAPI and with antibodies to p-Y705, S727 and CD138, after which they were visualized by ImageStream. Results for representative cells are shown. (BF = brightfield) (PNG 453 kb) [file 13402_2023_817_Fig13_ESM.png]

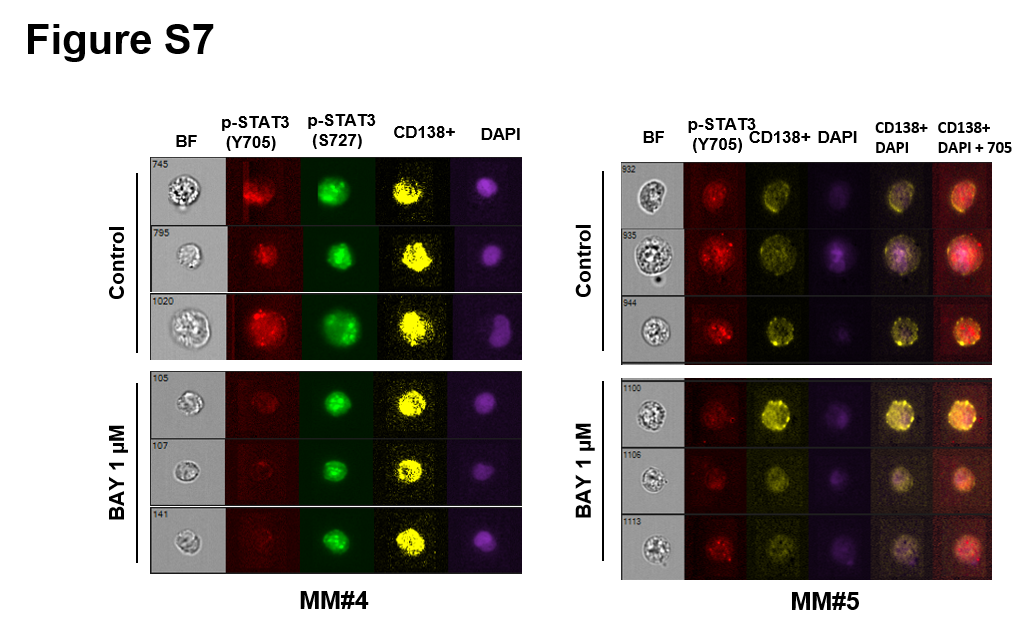

Supplement: Supplementary file 14 — High resolution image (TIF 598 KB) [file 13402_2023_817_MOESM7_ESM.tif]
